# Implications of *ZNF334* gene in lymph node metastasis of lung SCC: potential bypassing of cellular senescence

**DOI:** 10.1186/s12967-024-05115-9

**Published:** 2024-04-18

**Authors:** Khalil Khashei Varnamkhasti, Mehdi Moghanibashi, Sirous Naeimi

**Affiliations:** 1 Department of Genetics, Faculty of Basic Sciences, Kazerun Branch, Islamic Azad University, Kazerun, Iran; 2 Department of Genetics, Faculty of Medicine, Kazerun Branch, Islamic Azad University, Kazerun, Iran

**Keywords:** Lung squamous cell carcinoma, Lymph node metastasis, Senescence, *ZNF334* gene, *TINAGL1* gene

## Abstract

**Background:**

The primary goal of this work is to identify biomarkers associated with lung squamous cell carcinoma and assess their potential for early detection of lymph node metastasis.

**Methods:**

This study investigated gene expression in lymph node metastasis of lung squamous cell carcinoma using data from the Cancer Genome Atlas and R software. Protein-protein interaction networks, hub genes, and enriched pathways were analyzed. *ZNF334* and *TINAGL1*, two less explored genes, were further examined through in vitro, ex vivo, and in vivo experiments to validate the findings from bioinformatics analyses. The role of *ZNF334* and *TINAGL1* in senescence induction was assessed after H2O2 and UV induced senescence phenotype determined using β-galactosidase activity and cell cycle status assay.

**Results:**

We identified a total of 611 up- and 339 down-regulated lung squamous cell carcinoma lymph node metastasis-associated genes (FDR < 0.05). Pathway enrichment analysis highlighted the central respiratory pathway within mitochondria for the subnet genes and the nuclear DNA-directed RNA polymerases for the hub genes. Significantly down regulation of *ZNF334* gene was associated with malignancy lymph node progression and senescence induction has significantly altered *ZNF334* expression (with consistency in bioinformatics, in vitro, ex vivo, and in vivo results). Deregulation of *TINAGL1* expression with inconsistency in bioinformatics, in vitro (different types of lung squamous cancer cell lines), ex vivo, and in vivo results, was also associated with malignancy lymph node progression and altered in senescence phenotype.

**Conclusions:**

*ZNF334* is a highly generalizable gene to lymph node metastasis of lung squamous cell carcinoma and its expression alter certainly under senescence conditions.

**Supplementary Information:**

The online version contains supplementary material available at 10.1186/s12967-024-05115-9.

## Introduction

Lung cancer is the most lethal form of cancer worldwide, with squamous cell carcinoma (SCC) of the lung accounting for approximately 30% of cases [1]. The presence of lymph node metastasis (LNM) is a poor prognostic factor in lung SCC, with patients who have LNM experiencing worse overall survival [2, 3]. Although lymphadenectomy with microscopic evaluation is considered the most accurate method for identifying LNM, accumulating evidence suggests that it may still be incomplete [4]. This may be due to inadequate sampling during lung biopsies, as complete lymph node dissection can lead to complications, prolonged hospital stays, and/or increased mortality. Therefore, hidden metastases may go undetected on routine histological examinations [5].

Identifying potential tumor markers promoting lymph node metastasis is crucial to understanding the molecular characteristics underlying lung SCC [6] that can help with clinical diagnosis, prognostic assessment, and treatment strategies for such patients. Bioinformatics-based approaches have become important research tools in recent decades, enabling the identification of novel genes and key pathways involved in cancer progression and metastasis [7, 8].

Despite extensive data that has investigated the molecular mechanisms underlying lymph node metastasis in lung SCC [9–11], many of these studies have focused on a limited number of genes or pathways or have used small sample sizes without utilizing advanced bioinformatics methods and validation experiments to identify novel genes and pathways involved in this process. As a result, specific driver genes that are exclusive to lung SCC lymph node metastasis have yet to be identified, and understanding of the complex molecular mechanisms involved in lung SCC lymph node metastasis is still incomplete [12].

To overcome these limitations, we performed a comprehensive analysis of gene expression in a large cohort of lung SCC patients with and without lymph node metastasis, using bioinformatics approaches, to identify the most likely candidate genes specifically associated with lymph node metastasis in lung SCC. Due to the possibility that some putative genes proposed by bioinformatics may be incidental rather than deterministic, we also performed experimental methods to validate our findings for selected genes.

## Methods

### General information

**Fig. 1:**
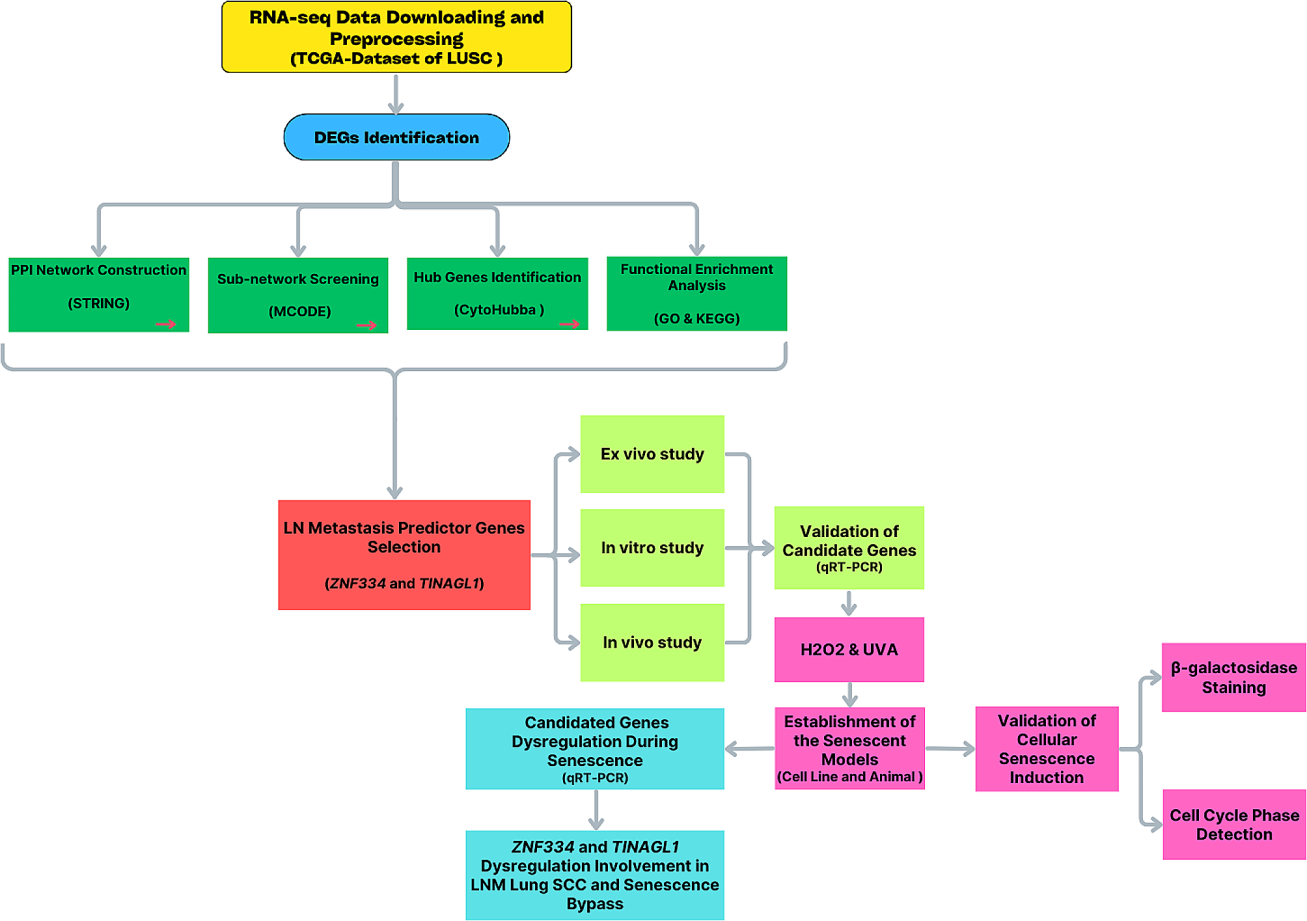
The flow chart of the study including data collection, processing, and analyses

This integrated bioinformatics-experimental study was conducted from July 10, 2022, to June 10, 2023, at the Central Laboratory, Isfahan University of Medical Sciences. All methods were performed in accordance with the relevant guidelines and regulations of the Islamic Azad University-Kazerun Branch Ethics Committee. A detailed overview of the research process is presented in Fig. 1.

### Data downloading and preprocessing

We searched for RNA sequencing (RNA-seq) data for both normal and cancer tissues for lung SCC lymph node involvement from the Cancer Genome Atlas (TCGA) database using the TCGA-LUSC dataset (https://portal.gdc.cancer.gov), and we obtained clinical data and gene expression profiles for 176 patients with N1, N2, and N3 lymph node involvement, 320 lymph node-negative (N0) patients, and 49 normal tissue samples.

To filter out highly and lowly expressed genes, we excluded genes with zero or very low counts in at least 10% of the samples based on counts per million (CPM). We calculated CPM using the cpm function in the edgeR package and normalized the data using the trimmed mean of m-values (TMM) method, and the “sva” package was used to remove the batch effect. Finally, we performed a log2 transformation using the limma package before conducting the differential expression test.

Considering the necessity of finding specific genes involved in lung SCC lymph node metastasis, N + status, we compared differentially expressed genes between the two biological states: N + vs. normal and N0 vs. normal by generalized linear modeling using the limma package in the R programming environment to test the differential expression analysis. We used a false discovery rate (FDR)-adjusted P value of < 0.05 as the threshold for screening differentially expressed genes (DEGs).

### PPI network construction, sub networks and hub genes screening

The search tool for retrieval of interacting genes (STRING) (https://string-db.org) database was applied to construct a protein-protein interaction (PPI) network based on the protein interaction data of the all N+-specific up- and down-regulated DEGs. The resulting network was visualized using the visualization software Cytoscape (version 3.9.0; National Institute of General Medical Sciences, Bethesda, MD, USA). Then, we investigated the subnets in the PPI network using the multi-contrast delayed enhancement (MCODE) plug-in (version 1.5.1) in the Cytoscape software to find subnets through the following clustering parameters: degree cutoff = 2, node score cutoff = 0.2, k-core = 2, and max. depth = 100. Moreover, to predict and explore important hub genes for N+-specific DEGs, the plug-in cytoHubba was used through 12 topological analysis methods, including MCC, DMNC, NMC, DEGREE, EPC, Bottle Neck, Eccentricity, Closeness, Radiality, Betweenness, Stress, and Clustering coefficient, with default parameters in Cytoscape. Hub genes and highly connected nodes (subnets) have a high probability of engaging in essential biological regulation and are often significantly associated with pathological conditions [13, 14].

### Functional enrichment analysis

We performed gene ontology (GO) enrichment analysis, which includes biological process (BP), molecular function (MF), and cellular component (CC) in the Kyoto Encyclopedia of Genes and Genomes (KEGG) using Enrichr (http://amp.pharm.mssm.edu/Enrichr/) [15] for all DEGs, hub genes and subnets.

### Clinical sample collection

We recruited eligible patients with pathologically confirmed lymph node metastasis who had received no prior treatment and informed consent was provided. We excluded patients with a previous cancer diagnosis, small cell lung cancer, secondary lung cancer, and metastasis to vital organs (distant metastasis). We collected lymphatic metastatic lung squamous cell carcinoma tissue samples from surgical and biopsy specimens between May 2020 and May 2023 from urban hospitals in Isfahan, Iran, including Alzahra, Sina, Khanevadeh and Milad Hospitals. Tumor and non-tumor tissues (located at least 5 cm from the tumor margin and confirmed by the pathologist to be free of obvious tumor cells) were immediately placed in a sterile 3 ml cold tube after removal from the body and frozen in liquid nitrogen at -196 °C.

### Cell culture

The SK-MES-1, NCI-H520, NCI-H2170 and MRC-5 cell lines were submerged in RPMI-1640 and DMEM medium (Bio-idea, Iran), respectively. The medium was supplemented with 10% (v/v) heat-inactivated fetal bovine serum (FBS) (Bio-idea, Iran) and 1:100 penicillin/streptomycin (Bio-idea, Iran). Cells were cultured in a humidified incubator at 37 °C and 5% CO2 (Binder, Germany), with the culture medium replaced with fresh medium three times a week. Cells were passaged using a trypsin/EDTA solution (Bio-idea, Iran) when they reached about 80% confluence. Six independent biological replicates were prepared on the cells of these cell lines derived from different passages for further analysis.

### Lung SCC tumor bearing C57BL/6J mouse model

Male pathogen-free C57BL/6J mice aged 4- to 6‐weeks were obtained from the Animal Laboratory of Isfahan University of Medical Sciences (Isfahan, IRAN). The mice were housed in groups of six per standard makrolon cage, under a 12-hour light-dark cycle, at room temperature 22 ± 2 °C and 50 ± 20% humidity. They were allowed access to food and water ad libitum. All animal studies were conducted in accordance with local guidelines for the care of laboratory animals at the Isfahan University of Medical Sciences. As many researchers have used the Lewis lung carcinoma cell line (LL/2) with high tumorigenic ability in C57BL and a higher rate of metastases than the original tumor line (LLC1) as a model for NSCLC [16–18], we also obtained this cell line from the national cell bank of the Pasteur Institute (Iran) and plated it as described above (i.e., DMEM/10% FBS at 37 °C in a 5% CO2/95% air atmosphere). LL/2 cells were harvested and resuspended in sterile PBS at a density of 1 × 10^6^ cells/100 µL as single‐cell suspensions. After intraperitoneal (IP) injection of a safe anesthetic (medetomidine, midazolam and butorphanol) in mice, on the left side of the chest, a skin incision and soft tissue dissection were made about 1 cm in order to expose intercostal space. Afterwards, LL/2 cells were injected into the left lung parenchyma using a 29‐G syringe needle, and the incised site was sutured. Mice were placed under a warm lamp until fully awake and were then housed in cages accommodating a normal diet for three weeks. All dissection activity completion was performed by an anatomist specialist expert. In parallel experiments, in the placebo groups, mice were treated intratracheally with PBS. Subsequently, mice were sacrificed, and prior to lung tissue collection, all accessories were cleaned with RNAse AWAY. Collected lungs were fixed with 10% neutral‐buffered formalin for histopathological and metastatic analyses and were kept in individually 1.5 ml Sterile Cryotube (Dominique Dutscher, Brumath, France) and frozen at -80 °C for molecular analysis.

### RNA extraction, complementary DNA synthesis, and quality assessment

The spun cell pellets (1∼2 × 10^6^ cells) and homogenized tissues (20–40 mg/human and animal tissue) were used for RNA extraction by the Pars Tous kit (Pars Tous, Iran, Cat No. A101231), applying the column-based RNA purification method to remove any contaminating genomic DNA. RNA concentrations were measured using a nanodrop spectrophotometer (Biochrom WPA Biowave II, UK). The purity of RNA was assessed by measuring the optical density (OD), and an OD 260/280 ratio higher than 1.8 is considered suitable for high-quality RNA. RNA integrity was also evaluated by performing electrophoresis on a 1% agarose gel, which revealed the presence of 18 S and 28 S rRNA bands. Complementary DNA (cDNA) synthesis was performed immediately after total RNA isolation using a cDNA synthesis kit (Pars Tous, Iran, Cat No. A101161). The cDNA synthesis kit contains 2X buffer mix solutions, including RT buffer, 1 mM dNTP mix, 8 mM MgCl2, oligo d(t)16, stabilizer, and enzyme mix, including thermostable Hminus MMLV, RNase inhibitor, and stabilizer. To assess the quality of cDNA, quantitative PCR (qPCR) was performed on an aliquot of cDNA from each sample using a housekeeping gene.

### Verification by qRT-PCR

Validation of RNA-seq results for selected genes was characterized by qRT-PCR analysis in vitro, ex vivo, and in vivo. Exon-exon junctions (excluding the *TINAGL1* gene) and common splice variants were applied for human PCR primer design by NCBI with the following sequences: *ZNF334*, forward 5`-GAGGAATGGCAGCAACTGGA-3’, reverse 5’-AAGGCATCATCAATGTCTGGGT-3’; *TINAGL1*, forward 5’-TGGGAGGCCAGAGAGATACC-3’, reverse 5’-TTGGCCGCAGTCCAGTATTT-3’ (Metabion, Germany). Mouse forward and reverse PCR primers with the following sequences: *ZNF334*, forward 5`-AGAACGCCTGGAAATGGACA-3’, reverse 5’-ATCTGCGTGGTTCTGTCGTG-3’; *TINAGL1*, forward 5’-ACCCCTCACCAGGAGGC-3’, reverse 5’-CAGTAACAGGTGGCTCCCAG-3’ (Metabion, Germany), were designed to amplify all splice variants of the transcript. Real-time amplification based on the RealQ Plus 2x Master Mix Green with high ROX™ ran in 0.2 mL 8 tube-strips. Reactions were prepared in a total volume of 10 µl comprising: 2 µl cDNA, 0.025 µl of each 10 µM primer, 5 µl of RealQ Plus 2x Master Mix Green with high ROX™ (Ampliqon); and 4.5 µl RNase/DNase-free sterile water (Qiagen). A no-template control (NTC) was run for each primer. The thermal cycling conditions were set as follows: initial template denaturation at 95 °C for 15 min, followed by 40 cycles of denaturation at 95 °C for 20 s, and combined primer annealing/elongation temperature specific to individual human and mouse primers at 60 °C and 58 °C for 30 s, respectively. This cycle was followed by a melting curve analysis at 95 °C to 60 °C. *GAPDH* was used to normalize the gene expression data, and all qRT-PCRs were performed in duplicate for tissue samples and in triplicate for cell lines. Ct values are considered the raw data for subsequent assessments of gene expression.

### Biomarkers for discrimination LNM by ROC curve

To evaluate the diagnostic accuracy of lung SCC lymph node metastasis candidate causative genes in discriminating metastatic and non-metastatic lymph nodes according to their tissue expression, a receiver operating characteristic (ROC) curve was constructed, and the areas under the curve (AUC) > 0.70 with a p-value < 0.05 were considered to have diagnostic value and be statistically significant.

### Establishment of the senescent cell line and animal models

Two different senescence-inducing stimuli (non-ionizing radiation, i.e., Ultraviolet (UV) radiation and oxidative stress) were used to promote cellular senescence in vitro in the SK-MES-1, NCI-H520 and NCI-H2170 cell lines. Cellular senescence was also activated in vivo in animal models in response to UV-radiation. Then, we analyzed the expression of *ZNF334* and *TINAGL1* genes in senescent cells established to provide a general view of expression of these genes and their related role in the cellular senescence phenomenon.

#### Cellular model of oxidative stress-induced senescence

Based on the hypothesis that hydrogen peroxide (H2O2) exposure would increase the number of senescent cells due to the accumulation of reactive oxygen species, SK-MES-1, NCI-H520 and NCI-H2170 cells were exposed to H2O2. H2O2 treatment in SK-MES-1, NCI-H520 and NCI-H2170 cell lines was performed in six independent biological replicate cultures in a 12-well plate (Nest Scientific, USA) either left untreated in medium (0µM) or treated according to the experimental groups, 25µM and 50µM (designed as sub-lethal doses of H2O2 following a pilot MTT test according to the standard protocol of the manufacturer of kit (thiazolyl blue tetrazolium bromide (Sigma-Aldrich Chemie, Taufkirchen, Germany) and a previously described protocol [19]) for 2 h [20]. The control group was considered untreated; however, the placebo control groups received the same sort of treatment at the exact same dosage as those in the experimental groups. Following inducing senescence, fresh RPMI medium supplemented with 1% FBS and antibiotics were added to the SK-MES-1, NCI-H520 and NCI-H2170 cells after being twice washed with phosphate-buffered saline (Bio-idea, Iran) for an additional 72-hour incubation.

#### Cellular model of ultraviolet radiation stress-induced senescence

Target SK-MES-1, NCI-H520 and NCI-H2170 cells acquired the senescence phenotype post-irradiation with a total dose of 8 J/cm^2^ of a UVA light (Philips lamp, emission spectrum 320 to 400 nm) twice a day, 20 min per session with 5-minute darkness intervals to minimize heat buildup, for 3 days. Prior to irradiation, different doses of UVA were tested, and this non-cytotoxic dose was chosen with more than 80% of viable cells detected after exposure. Moreover, the intensity and dose of energy emitted by the UVA light source were measured using an UV radiometer (Apollo 3.0, Labino, Sweden), and to prevent UVA absorption by factors within the growth medium, cells were submerged under a thin layer of PBS, and the cell plates were positioned at a 10 cm distance from the light source.

#### Murine model of ultraviolet radiation stress-induced senescence

The lung SCC tumor-bearing C57BL/6J mouse models were treated with UVA radiation. The head and lower back part of the body were the non-irradiated parts of the mice, wrapped with aluminum foil to fully block the UV light. The mice were then placed and fixed laterally in a self-made UV irradiation box for left lung irradiation. The hair of the irradiated part (a circular area of dorsal hair) of the mouse skin was removed a day before UV exposure. Throughout the daily UV irradiation cycle (2.2 kJ/m^2^ for 30 s on the shaved backs on seven consecutive days), no serious UV damage such as skin breaking or bleeding was observed. In parallel experiments, in the placebo groups, mice were exposed to the same dosage and time frame of UV delivery. Subsequently, mice were sacrificed, and all practices in tissue collection and sectioning procedures have been done with RNAse AWAY-cleaned accessories and microtomes. Collected lungs were fixed with 10% neutral-buffered formalin for histopathology staining, placed in a sterile cryotube (1.5 ml), and stored at − 80 °C for molecular analysis. Using a microtome, tissue specimens cut into sections of 5 μm were stained with hematoxylin and eosin for the cancer area and with 1X senescence-associated beta-galactosidase detection solution for the senescence area.

### Detection of lung SCC senescent phenotype

Two different traits, namely the measurement of senescence-associated β-galactosidase (SA-β-gal) activity and the halt of cell cycle progression, were employed to confirm the senescence-associated phenotype.

#### β-galactosidase staining

The SA-β-gal assay was carried out using a Cellular Senescence Assay Kit (Merck Millipore, USA, Catalog Number: KAA002) according to the manufacturer’s instructions. Briefly, at the end of the senescence-induction incubation period in culture, a medium containing the floating and loosely adherent cells was aspirated off, and the cells were rinsed with 1X PBS. Fifteen-minute incubations were performed at room temperature in a fixative solution (10 µL fixing solution in 990 µL PBS/well). After removing the fixing solution by washing twice with 1X PBS, cells were stained with the prepared 1X senescence-associated beta-galactosidase detection solution for 4 h in a dry incubator without CO2 and protected from light. Cellular senescence was visualized by phase contrast microscopy at 200× magnification.

For histochemical detection of SA-β-gal activity, slides with fresh tissue sections were fixed in SA-β-gal staining fix solution for 15 min at room temperature after rinsing the tissue to remove excess residual blood. Then sections were washed three times with PBS and incubated overnight with SA-β-gal staining solution at 37 °C without CO2. Finally, stained slides were viewed by microscope at 200× magnification and 200× for illustrations (phase contrast microscope, Zeiss, Germany).

#### Assaying cell cycle status

As assessing cell cycle distribution and proliferation is important for studying cell senescence, we analyzed cell cycle status post-observation of the pattern of SA-β-gal activity through propidium iodide (PI) staining using flow cytometry. For evaluation of cell cycle arrest, trypsin-digested SK-MES-1, NCI-H520 and NCI-H2170 cells and generated single-cell suspension (1 × 10^6^ cells) from homogenized tissue were collected and fixed in 70% (v/v) ethanol at 4 °C for 2 h and then incubated with 50 ul of RNase A for 30 min at 37 °C. Afterwards, cell pellets were stained with 1 ml of propidium iodide (BD Pharmingen, Heidelberg, Germany) staining solution (1 µg/mL PI in 1 mL PBS) at 4 °C for 30 min. FACSCalibur flow cytometer (BD Biosciences, USA) and BD FACSCantoTM system software (BD Biosciences, USA) were used to detect cell cycles. Doublets were excluded by creating a combination of same-channel bivariate plots utilizing area vs. height to ensure that only single cells were included in the analysis.

### Statistical analyses

All statistical analysis of RNAseq data was performed using R software (version 4.0.1). Non-parametric Wilcoxon (for two-group analyses) and Friedman tests (for multigroup analyses) recommended by Graph Pad Prism 8 statistical software (GraphPad Software, La Jolla, CA, USA) were used to determine statistical differences between experimental groups. Flow cytometry data analysis software, ModFit (Verity Software House) was used to plot all the cell cycle graphs. A p value < 0.05 was considered to be statistically significant.

## Results

### Identification of DEGs specified to lymph node metastasis

DEGs screening results indicated that 11,412 up- and 9148 down-regulated genes were found in N + lung SCC samples compared with normal samples (Supplementary File 1, Fig. 2A), and 11,549 up- and 9164 down-regulated genes were found in N0 samples compared to normal samples (Supplementary File 2, Fig. 2B). The Venny diagram showed that 611 up-regulated (Supplementary File 3, Fig. 2C) and 339 down-regulated (Supplementary File 4, Fig. 2D) genes are specific to the N + status of lung SCC.

**Fig. 2:**
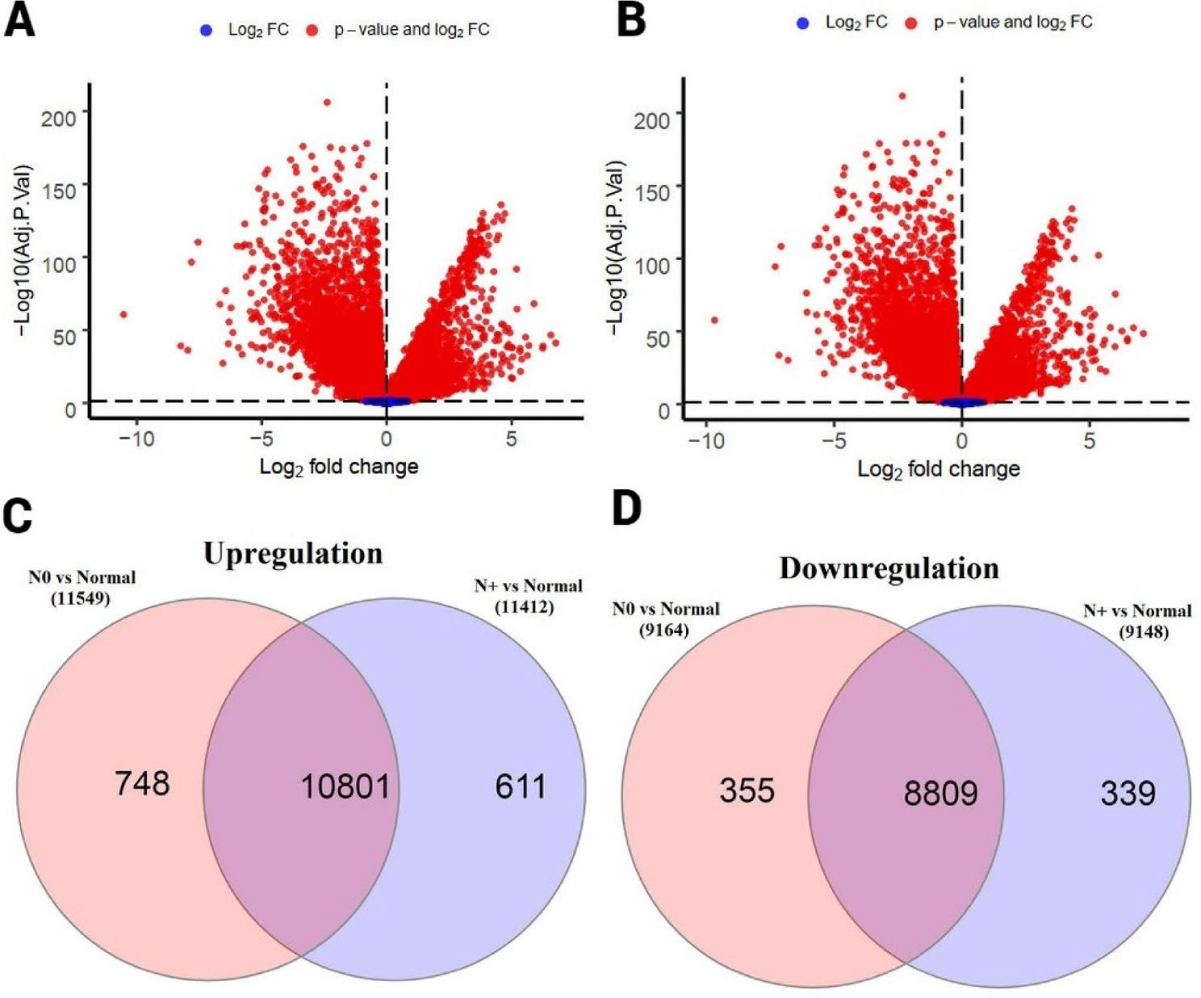
Identification of N+-specific DEGs. (**A**) Volcano plots reflecting significant DEGs in N + samples compared to normal samples; (**B**) Volcano plots reflecting significant DEGs in N0 samples compared to normal samples (**C**) Venn diagram of 611 up-regulated N+-specific genes; (**D**) Venn diagram of 339 down-regulated N+-specific genes. FDR < 0.05 is considered as a threshold

### Identification of PPI network, MCODE modules and hub genes

**Fig. 3:**
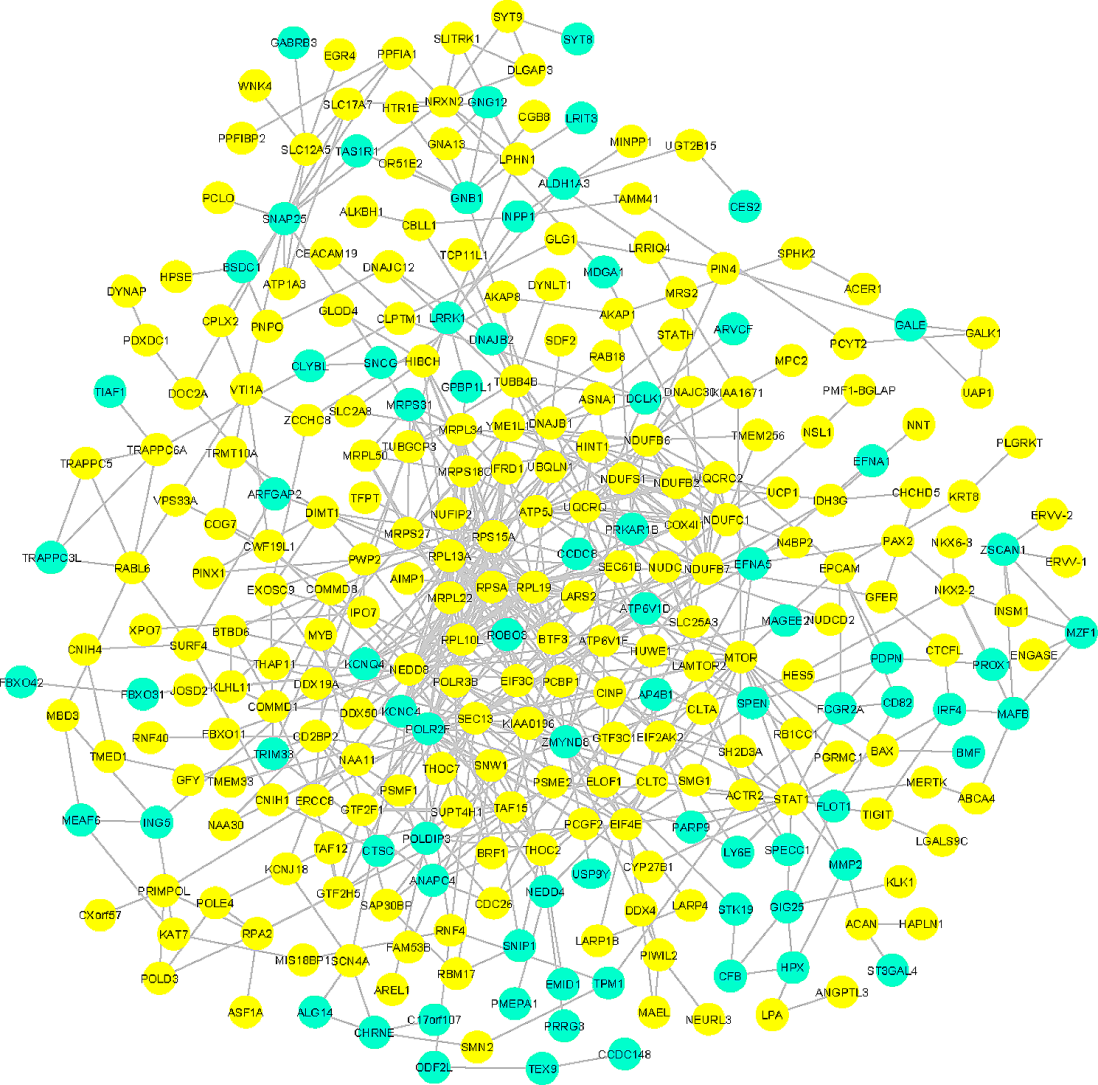
The overview of the protein-protein interaction network generated from all N+-specific DEGs processed using the STRING plug-in of the Cytoscape program (version 3.9.0). The circles represent genes (nodes), and connections between circles (edges) represent interactions. Upregulated genes have been depicted in yellow, while downregulated genes have been displayed in blue. Number of nodes: 345; number of edges: 713; average node degree: 8, expected number of edges: 3, avg. local clustering coefficient: 0.120; and PPI enrichment p-value: <1.0e-16

**Fig. 4:**
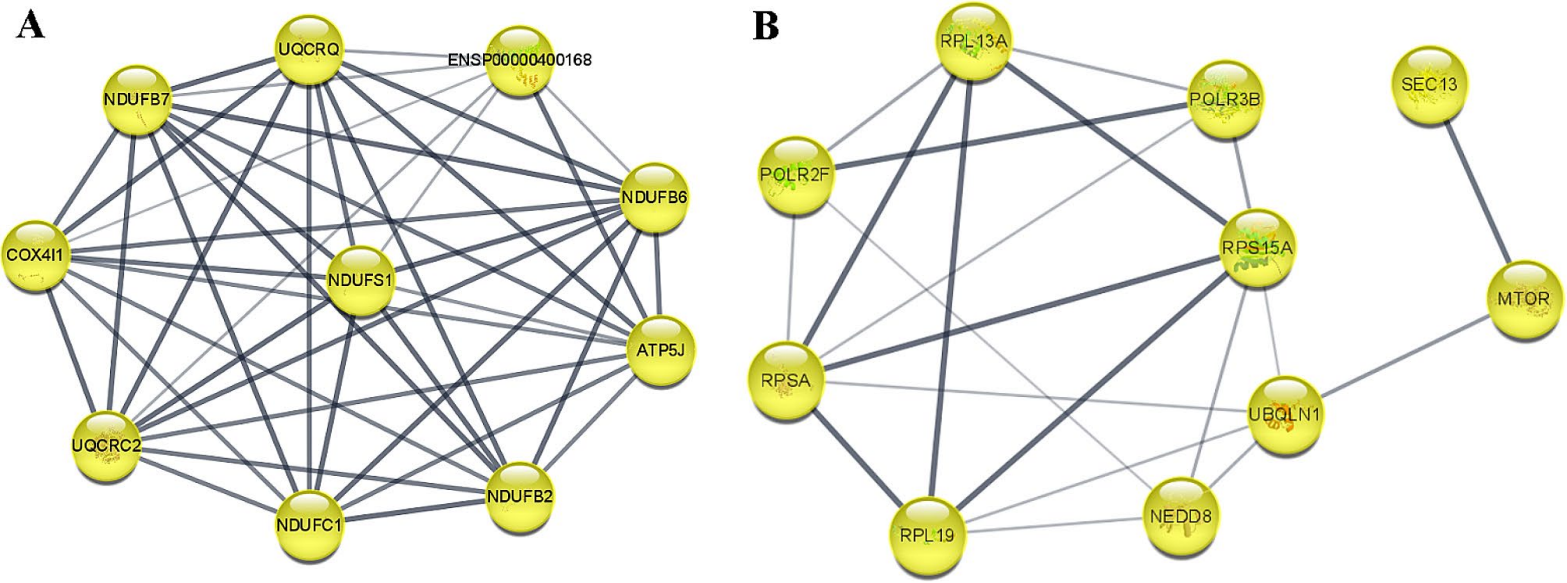
Best subnet from a highly interconnected region of N+-specific DEGs and PPI for 10 hub N+-specific DEGs that have been extracted using MCODE and cytoHubba analyses. Module as subnetwork (**A**) contains 9 nodes, 29 edges, average node degree: 6.44, expected number of edges: 1, avg. local clustering coefficient: 0.972, PPI enrichment p-value: < 1.0e-16; Network for hub genes (**B**) contains 10 nodes, 24 edges, average node degree: 4.8, expected number of edges: 8, avg. local clustering coefficient: 0.781, PPI enrichment p-value: 3.06e-06

Based on the 950 N+-specific DEGs, we obtained a PPI network consisting of 345 nodes and 713 edges except for self-interactions, non-physical interactions (disconnected nodes), and redundancies (PPI enrichment p-value: < 1.0e-16) (Fig. 3). We then employed this extended protein-protein interaction network to select smaller, densely connected gene subnetworks with high performance measures, as these subnetworks are expected to show high biological relevance, using MCODE plugin cytoscape software. The best subnetwork of the N+-specific DEG-PPI network with the highest MCODE score (9.55) contains 9 nodes and 29 edges (PPI enrichment p-value: < 1.0e-16) (Fig. 4A) including *COX4I1, NDUFS1, NDUFC1, NDUFB2, UQCRC2, UQCRQ, NDUFB6, ATP5J (ATP5PF), NDUFB7* and the *ENSP00000400168 (ATP5MF-PTCD1)* genes (Table 1).

**Table 1:** Identified the best subnetwork corresponding genes

Gene Name	N0 vs. Normal (LogFC)	N0 vs. Normal (FDR)	N + vs. Normal (logFC)	N + vs. Normal (FDR)	LogFC N+/ N0 Ratio
*NDUFB7*	0.216247648	0.061668628	0.367994924	0.002300595	1.7017291397
*UQCRC2*	0.099645297	0.260477817	0.203197936	0.02638368	2.0392125079
*COX4I1*	0.176077577	0.085246657	0.248263819	0.020432064	1.4099683971
***NDUFC1***	0.005794499	0.949396432	0.21442398	0.014598299	**37.0047488143**
***NDUFB6***	0.087348083	0.47744185	0.340986288	0.006036198	**3.903763841**
*UQCRQ*	0.173198783	0.103709276	0.285112753	0.01021194	1.6461591015
***NDUFB2***	0.026992126	0.782106506	0.204394875	0.034047822	**7.5723888885**
*NDUFS1*	0.091951633	0.246583612	0.188908107	0.021231973	2.0544290605
*ATP5PF*	0.186177324	0.051236781	0.244233098	0.014748681	1.3118305321
*ATP5MF-PTCD1*	0.030909263	0.071669537	0.044873335	0.01245501	1.4517762847

Afterwards, using plug-in cytoHubba in cytoscape software, we found 10 N+-specific hub genes, including *POLR2F*, *RPL19*, *RPL13A*, *MTOR*, *NEDD8*, *RPSA*, *POLR3B*, *SEC 13*, *RPS15A*, and *UBQLN1*, which were common in at least 5 methods of 12 cytoHubba different topological algorithms (Table 2). We also obtained a PPI for 10 hub genes using STRING (Fig. 4B) with 10 nodes and 24 edges (PPI enrichment p-value: 3.06e-06).

**Table 2:** Hub genes ranked by 12 different algorithms of cytohubba

MCC	DMNC	NMC	DEGREE	EPC	Bottle neck
*ATP5PF* *ATP5MF-PTCD1* *NDUFB7* *NDUFB6* *UQCRC2* *NDUFB2* *NDUFS1* *NDUFC1* *COX4I1* *UQCRQ*	*ATP5MF-PTCD1* *NDUFB7* *MRPL34* *NDUFB6* *NDUFB2* *NDUFS1* *NDUFC1* *COX4I1* *RPL10L* *MRPL50*	***POLR2F*** *SNAP25* ***RPSA*** ***RPL19*** ***RPL13A*** *UQCRQ* *RPS15A* ***MTOR*** ***POLR3B*** *MRPL22*	***POLR2F*** *SNAP25* ***RPSA*** ***NEDD8*** ***RPL19*** ***RPL13A*** ***SEC 13*** ***RPS15A*** ***MTOR*** *MRPL22*	***POLR2F*** *ATP5PF* ***RPSA*** ***NEDD8*** ***RPL19*** ***RPL13A*** *UQCRQ* ***RPS15A*** ***POLR3B*** *MRPL22*	***POLR2F*** *SNAP25* ***NEDD8*** ***RPL19*** *EIF4E* ***RPL13A*** ***SEC 13*** ***MTOR*** *STAT1* ***POLR3B***
**Eccentricity**	**Closeness**	**Radiality**	**Betweenness**	**Stress**	**Clustering coeficient**
*PSME2* ***POLR2F*** *CCDC8* *PSMF1* ***RPL19*** ***RPL13A*** *ERCC8* ***RPS15A*** ***UBQLN1*** *MRPL22*	***POLR2F*** ***RPSA*** ***NEDD8*** ***RPL19*** ***RPL13A*** ***SEC 13*** ***RPS15A*** ***MTOR*** ***POLR3B*** ***UBQLN1***	***POLR2F*** ***RPSA*** ***NEDD8*** ***RPL19*** ***RPL13A*** ***SEC 13*** *LARS2* ***RPS15A*** ***MTOR*** ***UBQLN1***	***POLR2F*** *SNAP25* ***NEDD8*** ***RPL19*** ***RPL13A*** ***SEC 13*** *MMP2* ***MTOR*** ***POLR3B*** ***UBQLN1***	***POLR2F*** ***RPSA*** ***NEDD8*** ***RPL19*** ***RPL13A*** ***SEC 13*** ***RPS15A*** ***MTOR*** ***POLR3B*** ***UBQLN1***	*PPFIBP2* *MPC2* *INVS* *UCP1* *BTBD6* *PRIMPOL* *RPL41* *SMG1* *AKIRIN2* *AKIRIN1*

### Enriched function for PPI and subnetwork genes

No significant results were obtained when all specific DEGs associated with lymph node metastasis were examined for Go and KEGG analyses. However, GO but not KEGG enrichment analysis for subnetworks significantly showed that for CC, subnetwork genes involved in mitochondrial protein complexes and respiratory chains, mitochondrial membrane, and mitochondrial envelope (Table 3) are also significantly associated with BP, such as coupling of ETC (electron transport chain) and ATP synthesis (Aerobic Electron Transport Chain and Mitochondrial ATP Synthesis Coupled Electron Transport) (Table 3) as well as MF linked to NADH Dehydrogenase (also known as NADH oxidoreductase) and Oxidoreduction-Driven Active Transmembrane Transporter Activity (Table 3).

**Table 3:** GO enrichment analysis of Cellular Component, Biological Process and Molecular Function categories of MCODE genes

Cellular Component	Description	P-value	Adjusted p-value	Odds Ratio	Combined Score
1	Respiratory Chain Complex I (GO:0045271)	7.030e-12	4.921e-11	554.28	14234.30
2	Mitochondrial Respiratory Chain Complex I (GO:0005747)	7.030e-12	4.921e-11	554.28	14234.30
3	Respiratory Chain Complex III (GO:0045275)	0.00001235	0.00002469	555.03	6273.06
4	Mitochondrial Respiratory Chain Complex III (GO:0005750)	0.00001235	0.00002469	555.03	6273.06
5	Mitochondrial Respiratory Chain Complex IV (GO:0005751)	0.00003048	0.00005334	332.92	3461.84
6	Respiratory Chain Complex IV (GO:0045277)	0.00003830	0.00005958	293.72	2987.15
7	Mitochondrial Inner Membrane (GO:0005743)	8.018e-11	3.742e-10	126.16	2932.82
8	Organelle Inner Membrane (GO:0019866)	1.337e-10	4.678e-10	116.96	2659.16
9	Mitochondrial Membrane (GO:0031966)	1.126e-9	3.152e-9	85.18	1755.08
10	Mitochondrial Intermembrane Space (GO:0005758)	0.0004187	0.0005329	83.04	645.92
11	Mitochondrial Envelope (GO:0005740)	0.00004404	0.00006166	59.48	596.62
12	Organelle Envelope Lumen (GO:0031970)	0.0005036	0.0005876	75.47	573.09
13	Mitochondrial Matrix (GO:0005759)	0.1640	0.1766	6.16	11.14
14	Intracellular Organelle Lumen (GO:0070013)	0.3544	0.3544	2.49	2.58
**Biological Process**	Description	P-value	Adjusted p-value	Odds Ratio	Combined Score
1	Aerobic Electron Transport Chain (GO:0019646)	5.214e-19	7.324e-18	1328.67	55933.89
2	Mitochondrial ATP Synthesis Coupled Electron Transport (GO:0042775)	6.658e-19	7.324e-18	1285.68	53809.88
3	Cellular Respiration (GO:0045333)	3.390e-18	2.486e-17	1034.44	41611.25
4	Mitochondrial Electron Transport, NADH to Ubiquinone (GO:0006120)	2.614e-12	9.585e-12	688.31	18357.31
5	Aerobic Respiration (GO:0009060)	1.055e-13	5.804e-13	564.25	16859.87
6	Oxidative Phosphorylation (GO:0006119)	1.590e-13	6.997e-13	524.55	15458.46
7	NADH Dehydrogenase Complex Assembly (GO:0010257)	2.685e-11	6.564e-11	415.46	10112.50
8	Proton Motive Force-Driven Mitochondrial ATP Synthesis (GO:0042776)	2.685e-11	6.564e-11	415.46	10112.50
9	Mitochondrial Respiratory Chain Complex I Assembly (GO:0032981)	2.685e-11	6.564e-11	415.46	10112.50
10	Proton Motive Force-Driven ATP Synthesis (GO:0015986)	5.103e-11	1.123e-10	362.45	8589.64
11	Energy Derivation by Oxidation of Organic Compounds (GO:0015980)	3.852e-9	7.061e-9	341.04	6607.62
12	Mitochondrial Electron Transport, Ubiquinol to Cytochrome C (GO:0006122)	0.00001481	0.00002506	499.50	5554.53
13	Mitochondrial Respiratory Chain Complex Assembly (GO:0033108)	4.081e-10	8.163e-10	234.18	5062.76
14	ATP Synthesis Coupled Electron Transport (GO:0042773)	0.003495	0.005492	370.07	2093.27
15	Mitochondrial Electron Transport, Cytochrome C to Oxygen (GO:0006123)	0.007973	0.01169	147.96	714.91
16	Purine Ribonucleoside Triphosphate Metabolic Process (GO:0009205)	0.01589	0.02062	71.54	296.32
17	Apoptotic Mitochondrial Changes (GO:0008637)	0.01687	0.02062	67.20	274.29
18	ATP Metabolic Process (GO:0046034)	0.01687	0.02062	67.20	274.29
19	Regulation of Mitochondrial Membrane Potential (GO:0051881)	0.01933	0.02239	58.34	230.20
20	Purine Ribonucleotide Metabolic Process (GO:0009150)	0.04122	0.04535	26.65	84.98
21	Mitochondrion Organization (GO:0007005)	0.07813	0.08185	13.68	34.89
22	Apoptotic Process (GO:0006915)	0.1083	0.1083	9.67	21.50
**Molecular Function**	Description	P-value	Adjusted p-value	Odds Ratio	Combined Score
1	NADH Dehydrogenase (Ubiquinone) Activity (GO:0008137)	2.230e-12	5.228e-12	712.93	19127.14
2	Oxidoreduction-Driven Active Transmembrane Transporter Activity (GO:0015453)	6.053e-14	3.632e-13	623.19	18967.06
3	NADH Dehydrogenase (Quinone) Activity (GO:0050136)	2.614e-12	5.228e-12	688.31	18357.31
4	Cytochrome-C Oxidase Activity (GO:0004129)	0.005985	0.008978	201.81	1032.95
5	Active Monoatomic Ion Transmembrane Transporter Activity (GO:0022853)	0.01194	0.01433	96.46	427.12
6	Proton Transmembrane Transporter Activity (GO:0015078)	0.02228	0.02228	50.37	191.61

### Enriched function for hub genes

Also, we used KEGG and GO enrichment analyses to investigate and elucidate the functions of the hub genes. Enrichment analysis by the KEGG found the main significant pathway associated with RNA polymerase, the ribosome, and the mTOR signaling pathway (Table 4). According to the GO enrichment analysis results, hub genes clustered mainly in CC (Table 5), BP (Table 5), and MF (Table 5) involving three nuclear DNA-directed RNA polymerases (RNA polymerases I, II, and III, highlighting the importance of DNA-directed RNA polymerase III complex and DNA-directed RNA polymerase III activity in each category), ribosome, and tRNA transcription.

**Table 4:** KEGG enrichment analysis conducted to interpret biological meanings of hub genes

	Description	P-value	Adjusted p-value	Odds Ratio	Combined Score
1	RNA Polymerase	0.0001038	0.001834	172.08	1578.44
2	Ribosome	7.588e-7	0.00004021	85.87	1210.05
3	Coronavirus Disease	0.000003508	0.00009295	57.78	725.80
4	Cytosolic DNA-Sensing Pathway	0.0004323	0.005729	81.68	632.69
5	mTOR Signaling Pathway	0.002545	0.02248	32.63	194.90
6	Type II Diabetes Mellitus	0.02277	0.1157	49.25	186.27
7	Amyotrophic Lateral Sclerosis	0.0006524	0.006916	23.30	170.92
8	Protein Processing in Endoplasmic Reticulum	0.003126	0.02367	29.32	169.12

**Table 5:** GO enrichment analysis of cellular component, biological process and molecular function categories of hub genes

Cellular Component	Description	P-value	Adjusted p-value	Odds Ratio	Combined Score
1	RNA Polymerase III Complex (GO:0005666)	0.00003048	0.001006	332.92	3461.84
2	TORC2 Complex (GO:0031932)	0.003495	0.01648	370.07	2093.27
3	Cytosolic Small Ribosomal Subunit (GO:0022627)	0.0001826	0.001943	127.89	1100.92
4	Small Ribosomal Subunit (GO:0015935)	0.0001917	0.001943	124.69	1067.29
5	RNA Polymerase I Complex (GO:0005736)	0.005985	0.02195	201.81	1032.95
6	RNA Polymerase II, Core Complex (GO:0005665)	0.006979	0.02243	170.74	847.70
7	Cytosolic Large Ribosomal Subunit (GO:0022625)	0.0002944	0.001943	99.70	810.62
8	Large Ribosomal Subunit (GO:0015934)	0.0002944	0.001943	99.70	810.62
9	COPII Vesicle Coat (GO:0030127)	0.007476	0.02243	158.54	776.21
10	Ribosome (GO:0005840)	0.0004053	0.002229	84.45	659.65
**Biological Process**	Description	P-value	Adjusted p-value	Odds Ratio	Combined Score
1	Regulation of Pentose-Phosphate Shunt (GO:0043456)	0.002498	0.03953	555.17	3326.77
2	Positive Regulation of ER-associated Ubiquitin-Dependent Protein Catabolic Process (GO:1,903,071)	0.002997	0.03953	444.11	2580.41
3	Aggrephagy (GO:0035973)	0.002997	0.03953	444.11	2580.41
4	Cytoplasmic Translation (GO:0002181)	9.004e-8	0.00001855	149.07	2418.38
5	tRNA Transcription by RNA Polymerase III (GO:0042797)	0.003495	0.03953	370.07	2093.27
6	Regulation of Wound Healing, Spreading of Epidermal Cells (GO:1,903,689)	0.003495	0.03953	370.07	2093.27
7	Regulation of Myeloid Leukocyte Differentiation (GO:0002761)	0.003495	0.03953	370.07	2093.27
8	Regulation of Oxidative Stress-Induced Cell Death (GO:1,903,201)	0.003495	0.03953	370.07	2093.27
9	tRNA Transcription (GO:0009304)	0.003994	0.03953	317.19	1751.86
10	Modification-Dependent Macromolecule Catabolic Process (GO:0043632)	0.003994	0.03953	317.19	1751.86
**Molecular Function**	Description	P-value	Adjusted p-value	Odds Ratio	Combined Score
1	RNA Polymerase III Activity (GO:0001056)	0.00002354	0.0003531	384.17	4094.01
2	RNA Polymerase III Type 3 Promoter Sequence-Specific DNA Binding (GO:0001006)	0.002997	0.01101	444.11	2580.41
3	RNA Polymerase III Cis-Regulatory Region Sequence-Specific DNA Binding (GO:0000992)	0.003495	0.01101	370.07	2093.27
4	DNA-directed 5’-3’ RNA Polymerase Activity (GO:0003899)	0.0001252	0.0009387	155.92	1401.11
5	RNA Polymerase II Activity (GO:0001055)	0.004990	0.01176	246.68	1307.49
6	RNA Polymerase I Activity (GO:0001054)	0.005488	0.01176	222.00	1155.57
7	Polyubiquitin Modification-Dependent Protein Binding (GO:0031593)	0.02619	0.04911	42.60	155.17
8	Ribosome Binding (GO:0043022)	0.04171	0.06951	26.33	83.66
9	Kinase Activity (GO:0016301)	0.05176	0.07765	21.04	62.31
10	RNA Binding (GO:0003723)	0.003668	0.01101	8.81	49.38

### Selection of genes for experimental validation

In the present study, we first reviewed the literature to identify unreported genes among subnetwork and hub genes as candidates for experimental validation. Intriguingly, we found that not only 10 *COX4I1, NDUFS1, NDUFC1, NDUFB2, UQCRC2, UQCRQ, NDUFB6, ATP5J (ATP5PF), NDUFB7* and the *ENSP00000400168 (ATP5MF-PTCD1)* genes of our obtained PPI subnet but also all hub genes *POLR2F*, *RPL19*, *RPL13A*, *MTOR*, *NEDD8*, *RPSA*, *POLR3B*, *SEC 13*, *RPS15A*, *BQLN1* have been presented and verified in previous studies (Supplementary file 5). Then, by applying the FDR-adjusted P value and logFC filtering on all N+-specific DEGs among the top up- and down-regulated genes (Supplementary File 6), some of them were selected as candidates for validation. These chosen candidates were also evaluated for existing literature. Our literature review indicated that more novel, unreported genes are down-regulated compared to top-up-regulated genes. We selected *ZNF334* (FDR-adjusted P value = 0.006742199, logFC = -0.570167591) and *TINAGL1* (FDR-adjusted P value = 0.009304419, logFC = -0.687630634) genes among the top down-regulated genes for the qRT-PCR assay.

### qRT-PCR validation for *ZNF334* and *TINAGL1* genes in vitro, ex vivo, and in vivo

Although cancer cell lines, contrary to tissue samples that are heterogenous and, through bearing an in vivo microenvironment, have a better match with the patient’s tumor profile, are often thought to be homogenous and fail to maintain the heterogeneity of the original tumor profile, the employment of these human cancer-derived cell lines as in vitro models for studying cancer biology and testing hypotheses in translational research would be valuable as long as they have equal value or relevance as tumor models [21, 22]. In this work, we recruited a rarely used and very low-passaged lung SCC cell lines from the Pasteur Institute of Iran-North Research Center branch (The Pasteur Institute of Amol) that had a closer resemblance to the original tumor profiles than the commonly used same cell line of the Pasteur Institute of Iran located in Tehran, which has grown for too many passages. Therefore, by setting this relative relevance between cell line and tumors being studied, the expression of *ZNF334* and *TINAGL1* was also quantified on cultured cells.

**Fig. 5:**
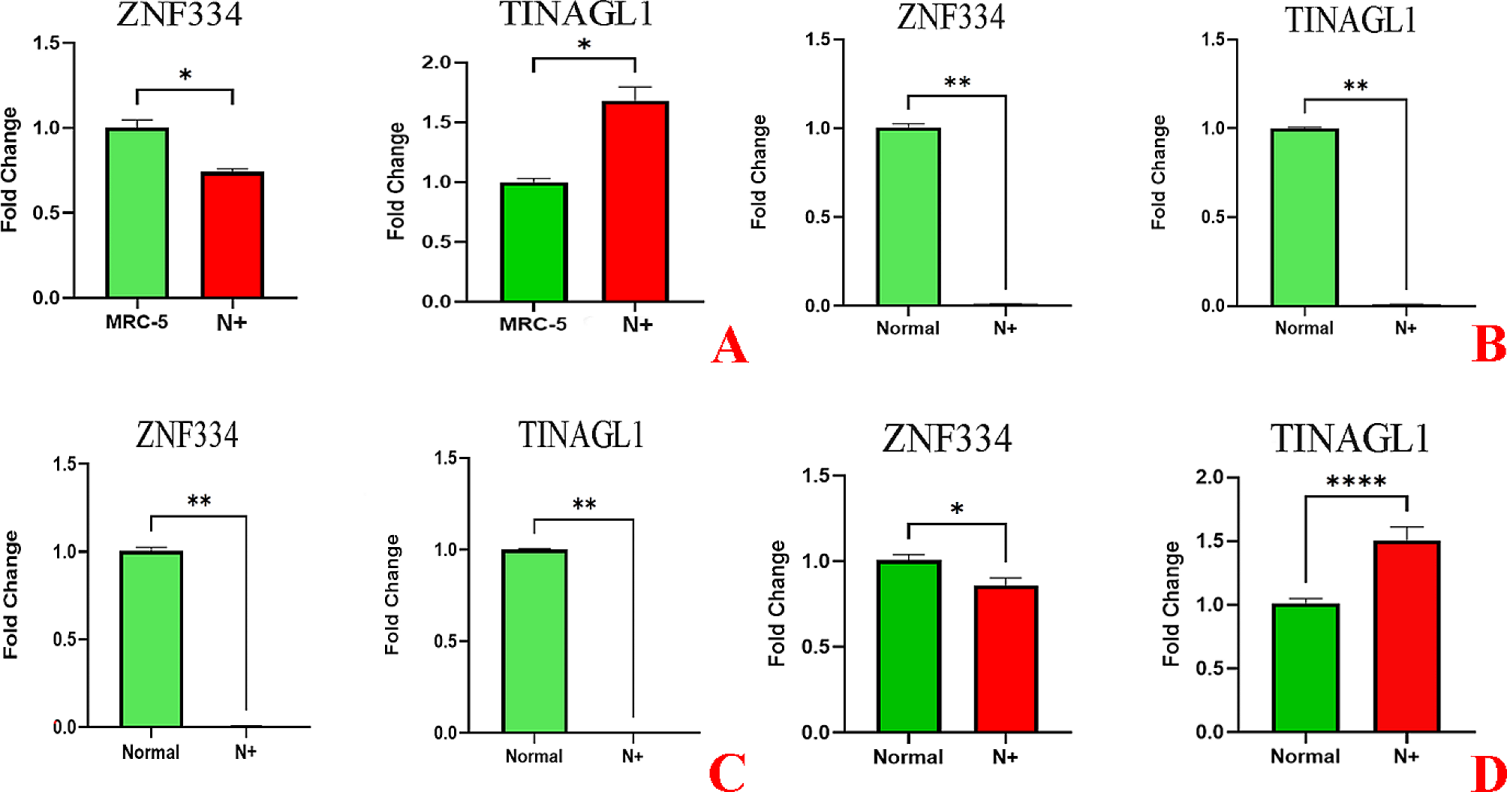
Gene expression analysis quantified by real-time PCR (qPCR) in N + lung SCC samples. **A-C** parts show qRT-PCR analysis of *ZNF334* and *TINAGL1* gene expression in the different types of lung squamous cancer cell lines ((**A**): SK-MES-1, (**B**): NCI-H520 and (**C**): NCI-H2170, respectively) and normal human lung cell line (MRC-5). Part D, shows qRT-PCR analysis of *ZNF334* and *TINAGL1* gene expression in the lung SCC lymphatic metastatic tissues and lung non-tumor tissues. Gene expression levels were normalized to the housekeeping gene (*GAPDH*). Data are expressed using the 2^−∆∆Ct^ method. Non-parametric Wilcoxon test was carried out to evaluate statistical differences between groups. Differences were considered significant at *p* < 0.05

Our quantitative in vitro analysis in the lung SCC cell lines showed that the expression of *ZNF334* gene was significantly downregulated (p value _SK-MES-1_ 0.0313, p value _NCI-H520_ 0.0078 and p value _NCI-H2170_ 0.0078) compared to the human normal lung cell line (MRC-5) (Fig. 5, A-C). The variable results obtained for the expression of *TINAGL1* gene on different types of lung squamous cancer cell lines. Although the significant upregulation of this gene is observed in SK-MES-1 cell line (p value 0.0313), its expression illustrated significantly downregulation in two other lung SCC cell lines (p value _NCI-H520_ 0.0078 and p value _NCI-H2170_ 0.0078) (Fig. 5, A-C). The result obtained from the in vitro validation was consistent with the results obtained from the bioinformatics analysis for the *ZNF334* gene; however, an inconsistency between the in vitro results for the *TINAGL1* gene and those obtained using bioinformatics analysis was observed.

We also validated these genes ex vivo by qRT-PCRs in the 24 paired samples of lymphatic metastatic lung squamous cell carcinoma and lung non-tumor tissues. Significantly decreased expression levels of *ZNF334* in N + tissues (p value 0.031) were shown to be consistent with bioinformatics and cell line results. However, a contradictory result was found with our bioinformatics results again, in which we detected significantly increased expression of the *TINAGL1* gene (p value 0.0001) in N + tissues in concordance with the cell line result (Fig. 5, D).

**Fig. 6:**
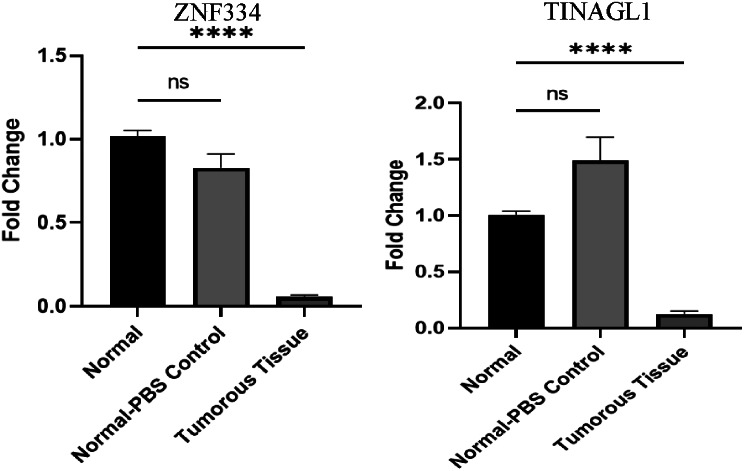
Decreased *ZNF334* and *TINAGL1* gene expression in mouse models of lung SCC. qRT-PCR analysis was performed using RNA prepared from 4- to 6‐weeks old male C57BL/6J mice (*n* = 6 per group). *GAPDH* was used as a reference gene. Data are expressed using the 2^−∆∆Ct^ method. Friedman test was carried out to evaluate statistical differences between groups. Differences were considered significant at *p* < 0.05

We next examined the expression of *ZNF334* and *TINAGL1* genes in lung specimens from C57BL/6J mice with LNM lung SCC tumorous lesions. *ZNF334* (p value < 0.0001) and *TINAGL1* (p value < 0.0001) mRNA expression revealed a marked decrease in the lung of SCC tumor-bearing C57BL/6J compared to normal lung tissue and placebo-controlled (Fig. 6). While the in vivo expression pattern for *ZNF334* was consistent with previous evidence from bioinformatics, in vitro and ex vivo expression pattern differences between mouse and human samples were observed for the *TINAGL1* gene.

### Diagnostic value of *ZNF334* and *TINAGL1* in human tissue

**Fig. 7:**
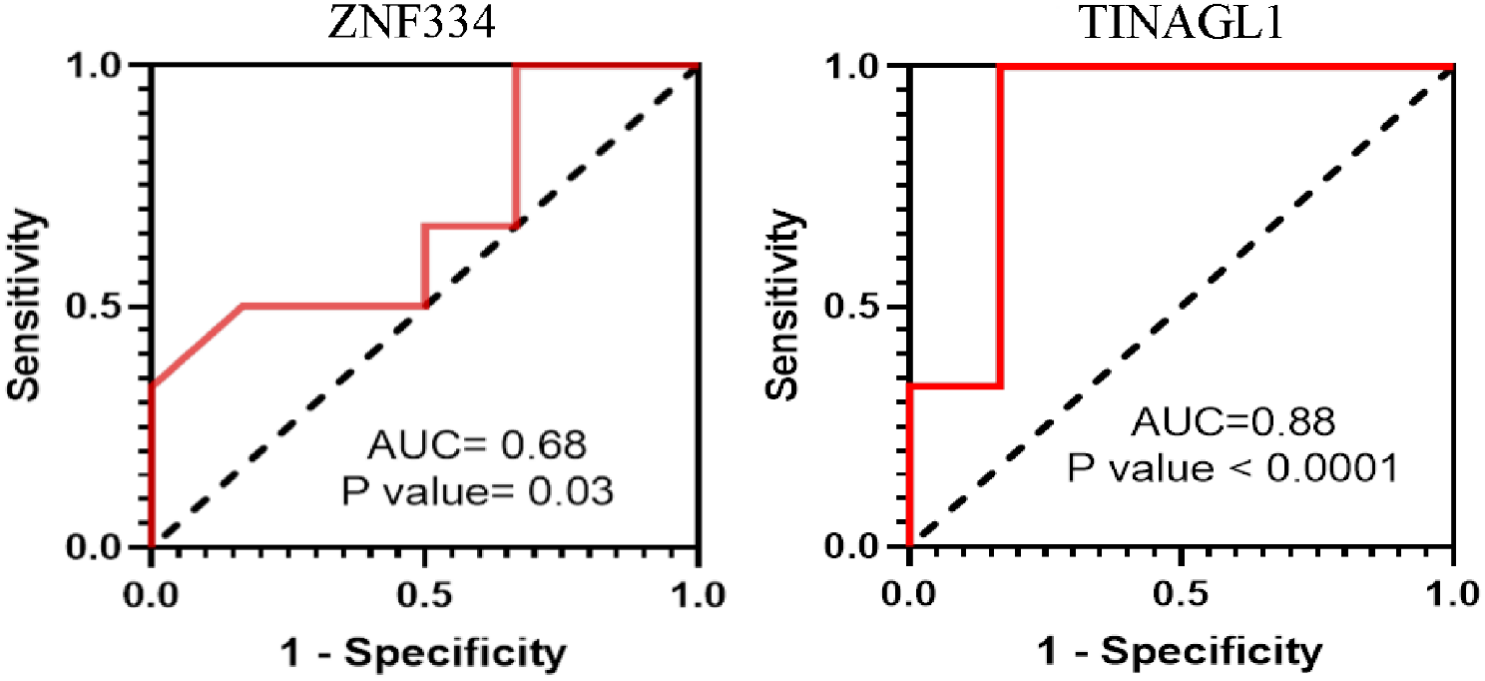
ROC curve analysis to test the validity of *ZNF334* and *TINAGL1* gene expression in distinguishing lung SCC lymph node metastasis involvement. p-value, specificity, and sensitivity were calculated. p-values smaller than 0.05 are considered significant. The potential biomarker with an AUC bigger than 0.8 is illustrated as an excellent biomarker

We also analyzed the diagnostic value of *ZNF334* and *TINAGL1* genes in the tissue samples of 24 patients with N + and normal tissue controls by ROC curves based on the area under the curve. The area under the curve was 0.68 for *ZNF334* and 0.88 for *TINAGL1* between lung SCC samples and lung non-tumor samples. The AUC of *ZNF334* was significant and higher than that of the random estimate (AUC 0.5), but could not be considered to have important diagnostic value in the ROC curves. However, *TINAGL1* had a better and more significant diagnostic value larger than 0.7 and suggested that it could be considered an excellent (AUC 0.8 to 0.9) potential biomarker (Fig. 7).

### Effect of senescence induction on *ZNF334* and *TINAGL1* expression in in vitro and in vivo model systems

Meanwhile, since zinc finger proteins have been implicated as transcription factors in cellular senescence and senescence is also associated with changes in ECM components and the role of senescent cells in invasiveness and lymphangiogenesis [23, 24], we examined the expression patterns of the *ZNF334* (a member of the C2H2 zinc finger family) and *TINAGL1* (a new member of the matricellular ECM proteins family) genes in the lung SCC senescence state [25, 26].

We have established cellular and murine models of the senescent phenotype in the SK-MES-1, NCI-H520 and NCI-H2170 cell lines and C57BL/6J mouse lung with LNM SCC tumorous lesions that have been triggered by senescence-inducers, including oxidative stress in the cellular model and ultraviolet radiation stress in both the cellular and murine models. Firstly, in order to evaluate whether exposure to H2O2 or UVA determines changes in cell viability in our models, we performed a dose-response determination procedure and estimated a level of toxicity by subjecting at different defined doses and looking for signs of cytotoxicity. The no-observed toxicity level was defined as a sub-lethal dosage at which there were no cytotoxic effects attributable to the H2O2 and UVA irradiation under test.

**Fig. 8:**
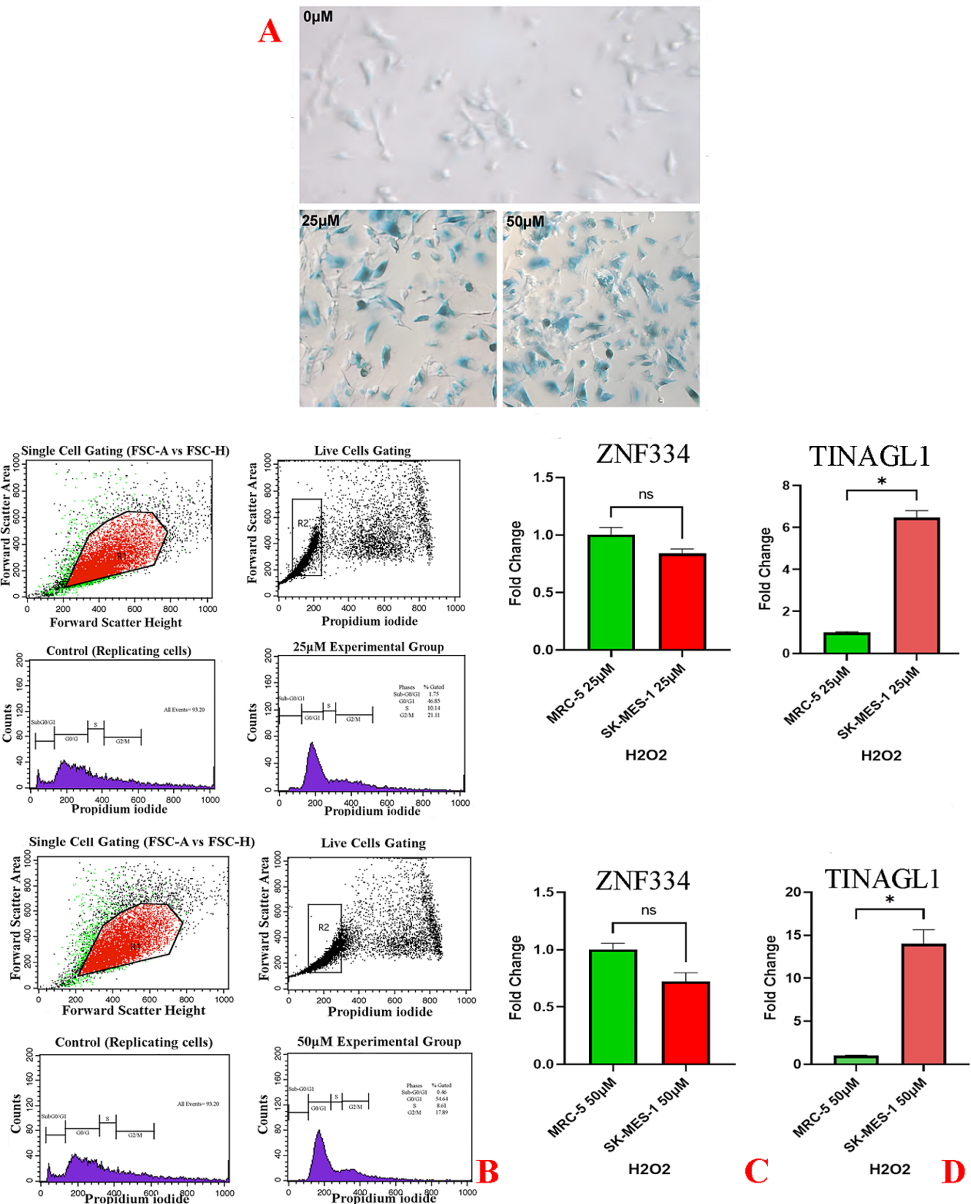
Cellular Model of Oxidative Stress-Induced Senescence in SK-MES-1 Cells. The cells were treated for 2 h without (0µM) or with 25µM and 50µM H2O2, and then treated with supplemented medium for an additional 72 h. **A** Cytochemical analysis of SA-β-gal activity; the lung SCC cells were stained for SA-β-gal and photographed with a phase contrast microscope at 200× magnification. **B** Flow Cytometry Gating for Single and Live Cells, Quarter 1: Forward Scatter Area vs. Forward Scatter Height; first gate that was used to exclude any doublets and clumps of multiple cells to eliminate skewed results, Quarter 2: Forward Scatter Area vs. Propidium iodide; gate that is used for live cells and gate out obvious debris (as dead cells often have varied areas and heights that can make data look sloppy), once the single cells of interest were the only cells in our analysis, Quarter 3: Control cells stained with propidium iodide show G1, S, and G2/M phase distributions. Quarter 4: The frequency of G1 senescent cells increased dramatically, and the frequencies of S and G2/M phases decreased significantly. Plots were made using ModFit. **C** Non-significant decreased *ZNF334* expression in senescent cells treated with H2O2. qRT-PCR analysis was performed using RNA prepared from senescent SK-MES-1 cells treated with 25µM and 50µM H2O2 concentrations. *GAPDH* was used as a reference gene. Data are expressed using the 2^−∆∆Ct^ method. Non-parametric Wilcoxon test was carried out to evaluate statistical differences between groups. Differences were considered significant when *p* < 0.05, **D** Significant increased *TINAGL1* expression (p value_25µM_ 0.0313, p value_50µM_ 0.0313) in senescent cells treated with H2O2. qRT-PCR analysis was performed using RNA prepared from senescent SK-MES-1 cells treated with 25µM and 50µM H2O2 concentrations. *GAPDH* was used as a reference gene. Data are expressed using the 2^−∆∆Ct^ method. Non-parametric Wilcoxon test was carried out to evaluate statistical differences between groups. Differences were considered significant at *p* < 0.05

**Fig. 9:**
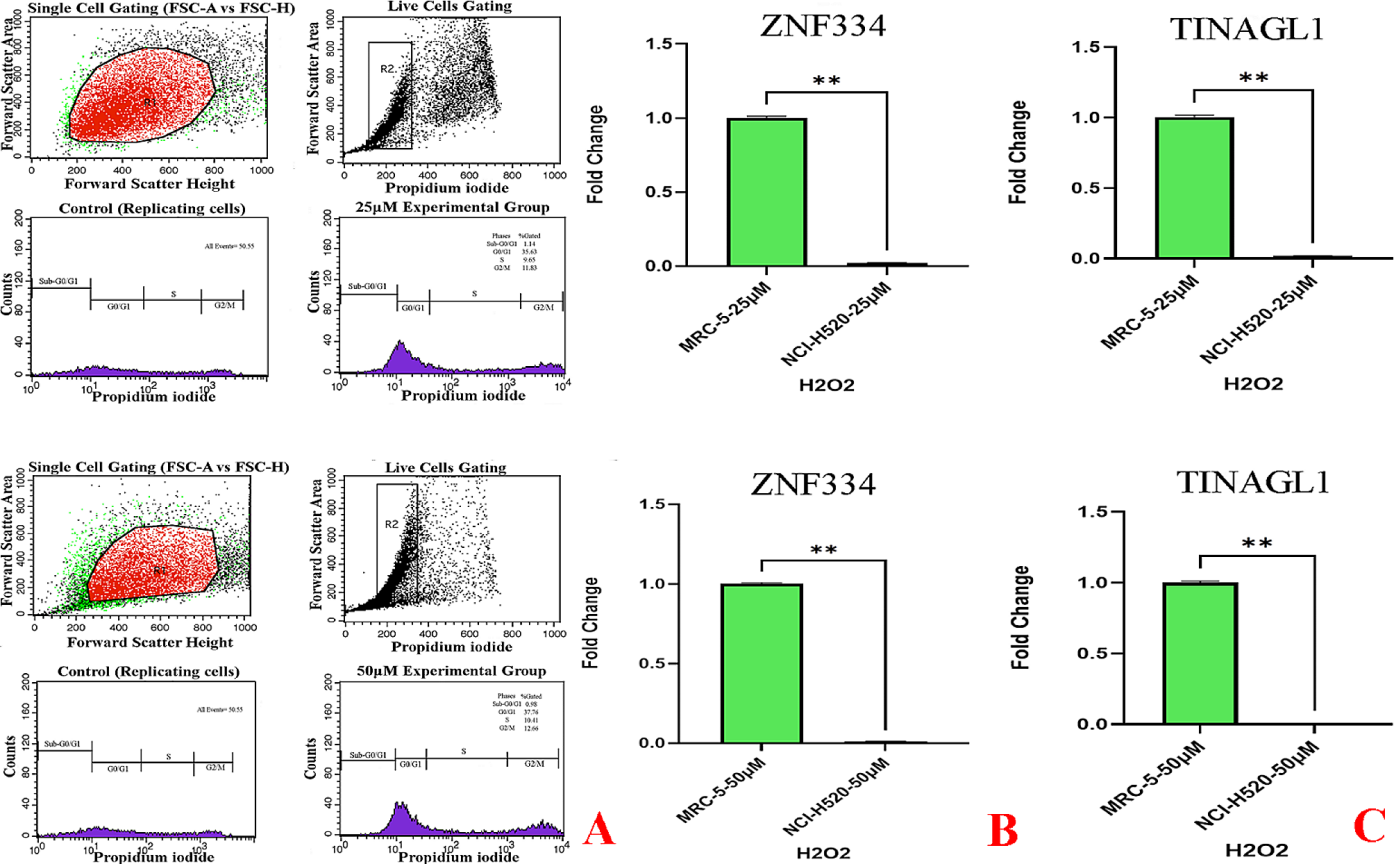
Cellular Model of Oxidative Stress-Induced Senescence in NCI-H520 Cells. The cells were treated for 2 h without (0µM) or with 25µM and 50µM H2O2, and then treated with supplemented medium for an additional 72 h. **A** Flow Cytometry Gating for Single and Live Cells, Quarter 1: Forward Scatter Area vs. Forward Scatter Height; first gate that was used to exclude any doublets and clumps of multiple cells to eliminate skewed results, Quarter 2: Forward Scatter Area vs. Propidium iodide; gate that is used for live cells and gate out obvious debris (as dead cells often have varied areas and heights that can make data look sloppy), once the single cells of interest were the only cells in our analysis, Quarter 3: Control cells stained with propidium iodide show G1, S, and G2/M phase distributions. Quarter 4: The frequency of G1 senescent cells increased dramatically, and the frequencies of S and G2/M phases decreased significantly. Plots were made using ModFit. **B** Significant decreased *ZNF334* expression (p value_25µM_ 0.0078, p value_50µM_ 0.0078) in senescent cells treated with H2O2. qRT-PCR analysis was performed using RNA prepared from senescent NCI-H520 cells treated with 25µM and 50µM H2O2 concentrations. *GAPDH* was used as a reference gene. Data are expressed using the 2^−∆∆Ct^ method. Non-parametric Wilcoxon test was carried out to evaluate statistical differences between groups. Differences were considered significant when *p* < 0.05, **C** Significant decreased *TINAGL1* expression (p value_25µM_ 0.0078, p value_50µM_ 0.0078) in senescent cells treated with H2O2. qRT-PCR analysis was performed using RNA prepared from senescent NCI-H520 cells treated with 25µM and 50µM H2O2 concentrations. *GAPDH* was used as a reference gene. Data are expressed using the 2^−∆∆Ct^ method. Non-parametric Wilcoxon test was carried out to evaluate statistical differences between groups. Differences were considered significant at *p* < 0.05

**Fig. 10:**
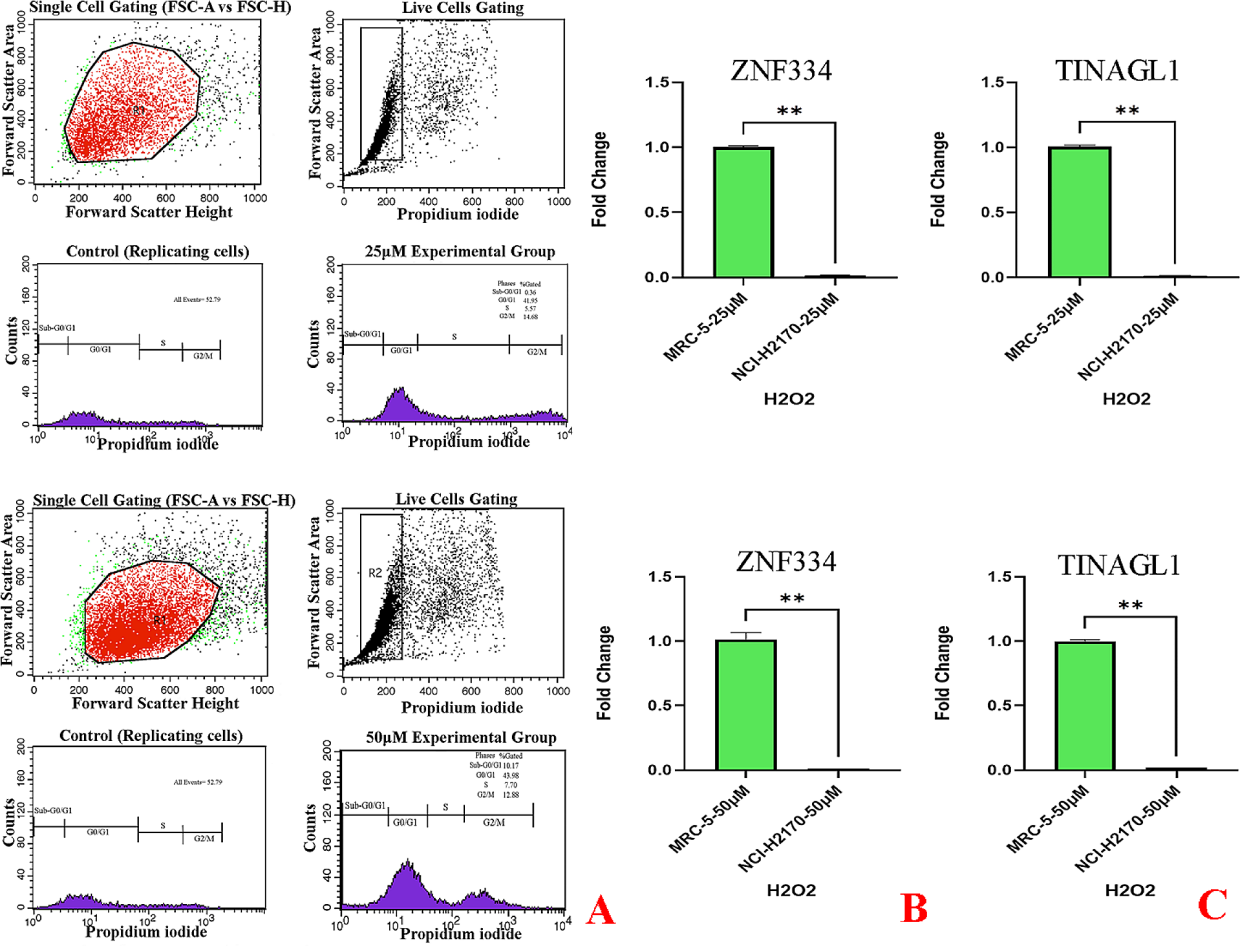
Cellular Model of Oxidative Stress-Induced Senescence in NCI-H2170 Cells. The cells were treated for 2 h without (0µM) or with 25µM and 50µM H2O2, and then treated with supplemented medium for an additional 72 h. **A** Flow Cytometry Gating for Single and Live Cells, Quarter 1: Forward Scatter Area vs. Forward Scatter Height; first gate that was used to exclude any doublets and clumps of multiple cells to eliminate skewed results, Quarter 2: Forward Scatter Area vs. Propidium iodide; gate that is used for live cells and gate out obvious debris (as dead cells often have varied areas and heights that can make data look sloppy), once the single cells of interest were the only cells in our analysis, Quarter 3: Control cells stained with propidium iodide show G1, S, and G2/M phase distributions. Quarter 4: The frequency of G1 senescent cells increased dramatically, and the frequencies of S and G2/M phases decreased significantly. Plots were made using ModFit. **B** Significant decreased *ZNF334* expression (p value_25µM_ 0.0078, p value_50µM_ 0.0078) in senescent cells treated with H2O2. qRT-PCR analysis was performed using RNA prepared from senescent NCI-H2170 cells treated with 25µM and 50µM H2O2 concentrations. *GAPDH* was used as a reference gene. Data are expressed using the 2^−∆∆Ct^ method. Non-parametric Wilcoxon test was carried out to evaluate statistical differences between groups. Differences were considered significant when *p* < 0.05, **C** Significant decreased *TINAGL1* expression (p value_25µM_ 0.0078, p value_50µM_ 0.0078) in senescent cells treated with H2O2. qRT-PCR analysis was performed using RNA prepared from senescent NCI-H2170 cells treated with 25µM and 50µM H2O2 concentrations. *GAPDH* was used as a reference gene. Data are expressed using the 2^−∆∆Ct^ method. Non-parametric Wilcoxon test was carried out to evaluate statistical differences between groups. Differences were considered significant at *p* < 0.05

**Fig. 11:**
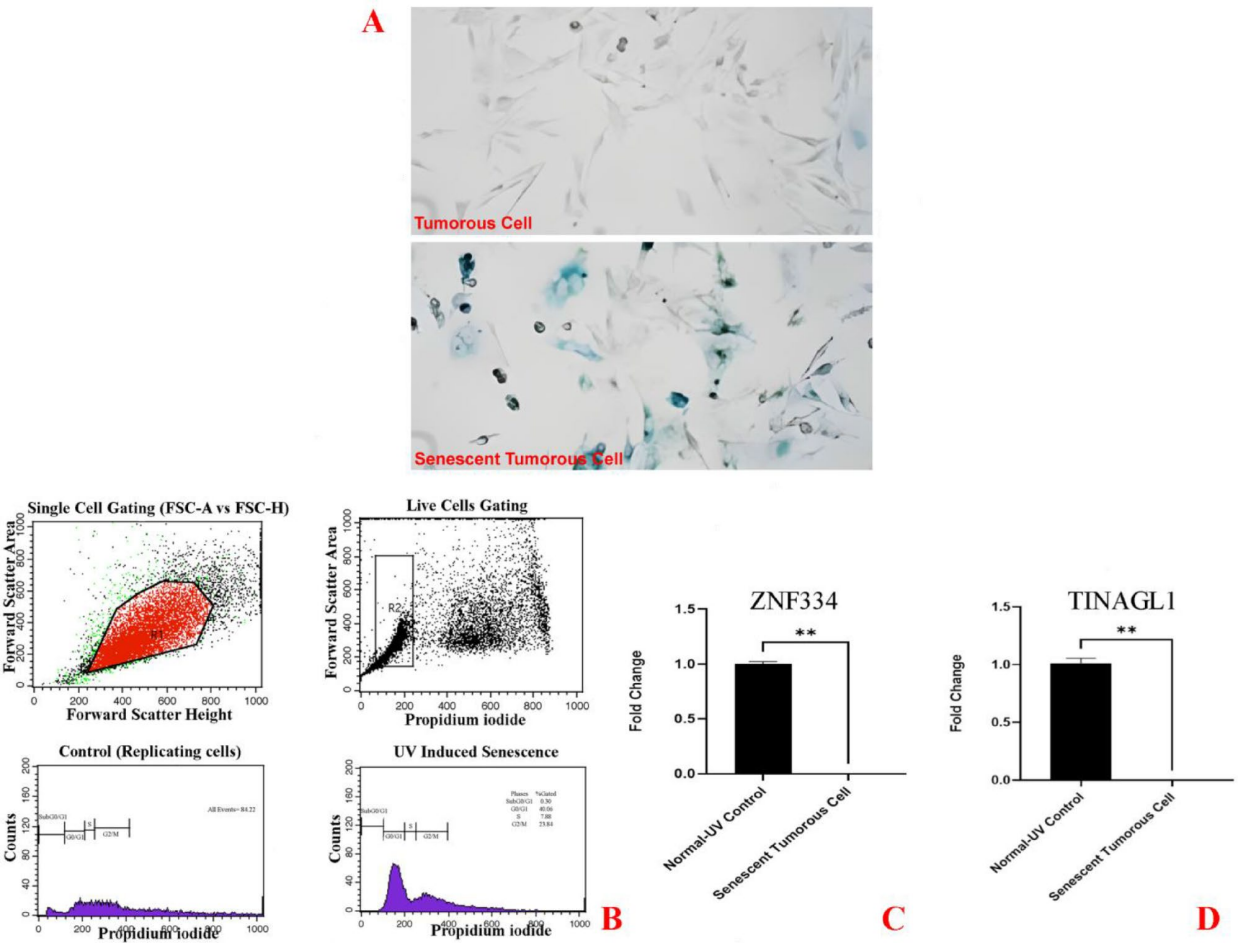
Cellular Model of Ultraviolet Radiation Stress-Induced Senescence in SK-MES-1 Cells. The cells were treated for 3 days (twice a day, 20 min per session) without (0 J/cm^2^) and with a total dose of 8 J/cm^2^ of UVA light. **A** Cytochemical analysis of SA-β-gal activity; the lung SCC cells were stained for SA-β-gal and photographed with a phase contrast microscope at 200× magnification. **B** Flow Cytometry Gating for Single and Live Cells, Quarter 1: Forward Scatter Area vs. Forward Scatter Height; first gate that was used to exclude any doublets and clumps of multiple cells to eliminate skewed results, Quarter 2: Forward Scatter Area vs. Propidium iodide; gate that is used for live cells and gate out obvious debris (as dead cells often have varied areas and heights that can make data look sloppy), once the single cells of interest were the only cells in our analysis, Quarter 3: Control cells stained with propidium iodide show G1, S, and G2/M phase distributions. Quarter 4: The frequency of G1 senescent cells increased dramatically, and the frequencies of S and G2/M phases decreased significantly. Plots were made using ModFit. **C** Significantly decreased *ZNF334* expression in senescent cells treated with UVA. qRT-PCR analysis was performed using RNA prepared from senescent SK-MES-1 cells treated with 8 J/cm^2^ of UVA light. *GAPDH* was used as a reference gene. Data are expressed using the 2^−∆∆Ct^ method. Non-parametric Wilcoxon test was carried out to evaluate statistical differences between groups. Differences were considered significant when *p* < 0.05. **D** Significantly decreased *TINAGL1* expression in senescent cells treated with UVA. qRT-PCR analysis was performed using RNA prepared from senescent SK-MES-1 cells treated with 8 J/cm^2^ of UVA light. *GAPDH* was used as a reference gene. Data are expressed using the 2^−∆∆Ct^ method. Non-parametric Wilcoxon test was carried out to evaluate statistical differences between groups. Differences were considered significant at *p* < 0.05

**Fig. 12:**
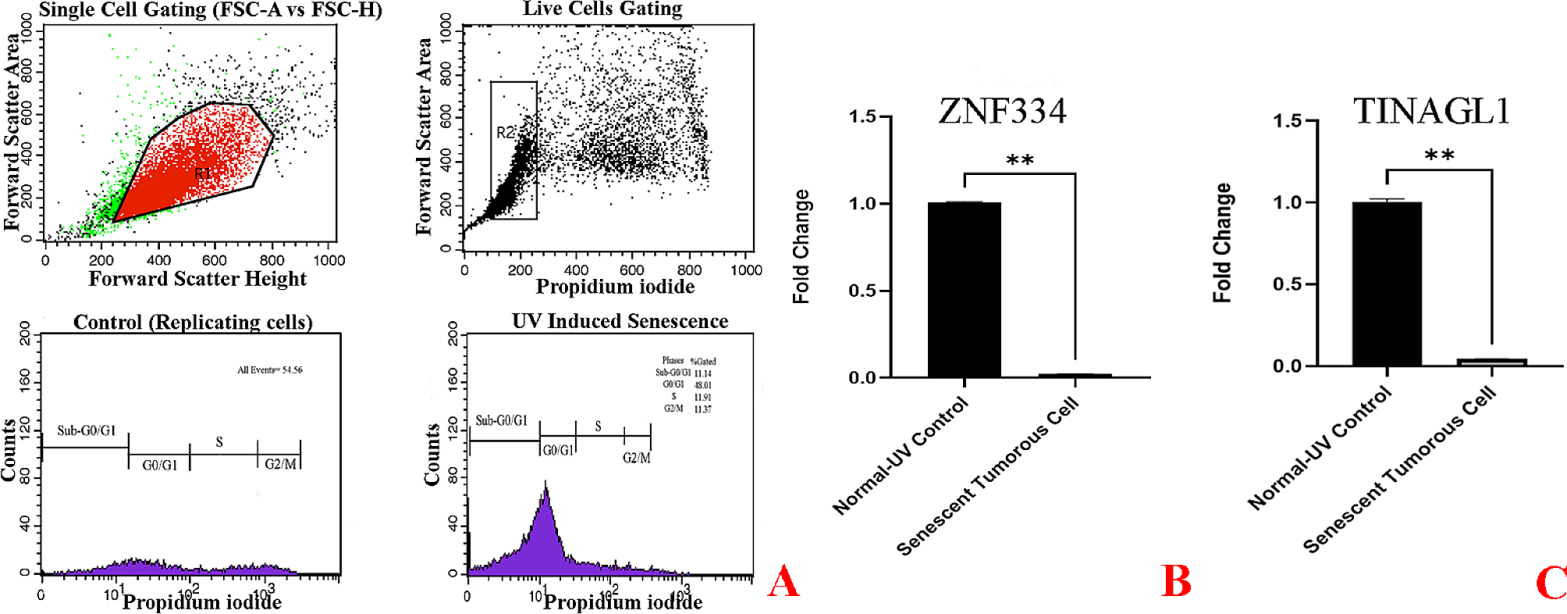
Cellular Model of Ultraviolet Radiation Stress-Induced Senescence in NCI-H520 Cells. The cells were treated for 3 days (twice a day, 20 min per session) without (0 J/cm^2^) and with a total dose of 8 J/cm^2^ of UVA light. **A** Flow Cytometry Gating for Single and Live Cells, Quarter 1: Forward Scatter Area vs. Forward Scatter Height; first gate that was used to exclude any doublets and clumps of multiple cells to eliminate skewed results, Quarter 2: Forward Scatter Area vs. Propidium iodide; gate that is used for live cells and gate out obvious debris (as dead cells often have varied areas and heights that can make data look sloppy), once the single cells of interest were the only cells in our analysis, Quarter 3: Control cells stained with propidium iodide show G1, S, and G2/M phase distributions. Quarter 4: The frequency of G1 senescent cells increased dramatically, and the frequencies of S and G2/M phases decreased significantly. Plots were made using ModFit. **B** Significantly decreased *ZNF334* expression in senescent cells treated with UVA. qRT-PCR analysis was performed using RNA prepared from senescent NCI-H520 cells treated with 8 J/cm^2^ of UVA light. *GAPDH* was used as a reference gene. Data are expressed using the 2^−∆∆Ct^ method. Non-parametric Wilcoxon test was carried out to evaluate statistical differences between groups. Differences were considered significant when *p* < 0.05. **C** Significantly decreased *TINAGL1* expression in senescent cells treated with UVA. qRT-PCR analysis was performed using RNA prepared from senescent NCI-H520 cells treated with 8 J/cm^2^ of UVA light. *GAPDH* was used as a reference gene. Data are expressed using the 2^−∆∆Ct^ method. Non-parametric Wilcoxon test was carried out to evaluate statistical differences between groups. Differences were considered significant at *p* < 0.05

**Fig. 13:**
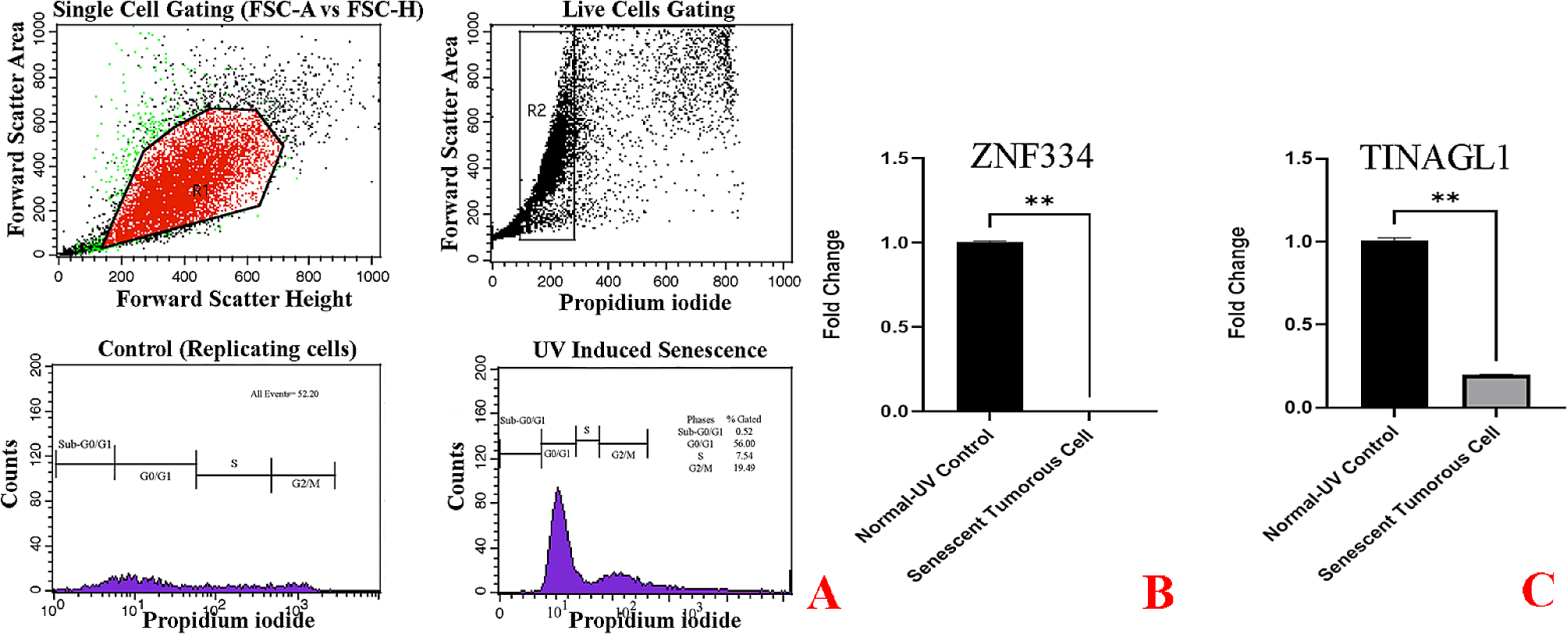
Cellular Model of Ultraviolet Radiation Stress-Induced Senescence in NCI-H2170 Cells. The cells were treated for 3 days (twice a day, 20 min per session) without (0 J/cm^2^) and with a total dose of 8 J/cm^2^ of UVA light. **A** Flow Cytometry Gating for Single and Live Cells, Quarter 1: Forward Scatter Area vs. Forward Scatter Height; first gate that was used to exclude any doublets and clumps of multiple cells to eliminate skewed results, Quarter 2: Forward Scatter Area vs. Propidium iodide; gate that is used for live cells and gate out obvious debris (as dead cells often have varied areas and heights that can make data look sloppy), once the single cells of interest were the only cells in our analysis, Quarter 3: Control cells stained with propidium iodide show G1, S, and G2/M phase distributions. Quarter 4: The frequency of G1 senescent cells increased dramatically, and the frequencies of S and G2/M phases decreased significantly. Plots were made using ModFit. **B** Significantly decreased *ZNF334* expression in senescent cells treated with UVA. qRT-PCR analysis was performed using RNA prepared from senescent NCI-H2170 cells treated with 8 J/cm^2^ of UVA light. *GAPDH* was used as a reference gene. Data are expressed using the 2^−∆∆Ct^ method. Non-parametric Wilcoxon test was carried out to evaluate statistical differences between groups. Differences were considered significant when *p* < 0.05. **C** Significantly decreased *TINAGL1* expression in senescent cells treated with UVA. qRT-PCR analysis was performed using RNA prepared from senescent NCI-H2170 cells treated with 8 J/cm^2^ of UVA light. *GAPDH* was used as a reference gene. Data are expressed using the 2^−∆∆Ct^ method. Non-parametric Wilcoxon test was carried out to evaluate statistical differences between groups. Differences were considered significant at *p* < 0.05

**Fig. 14:**
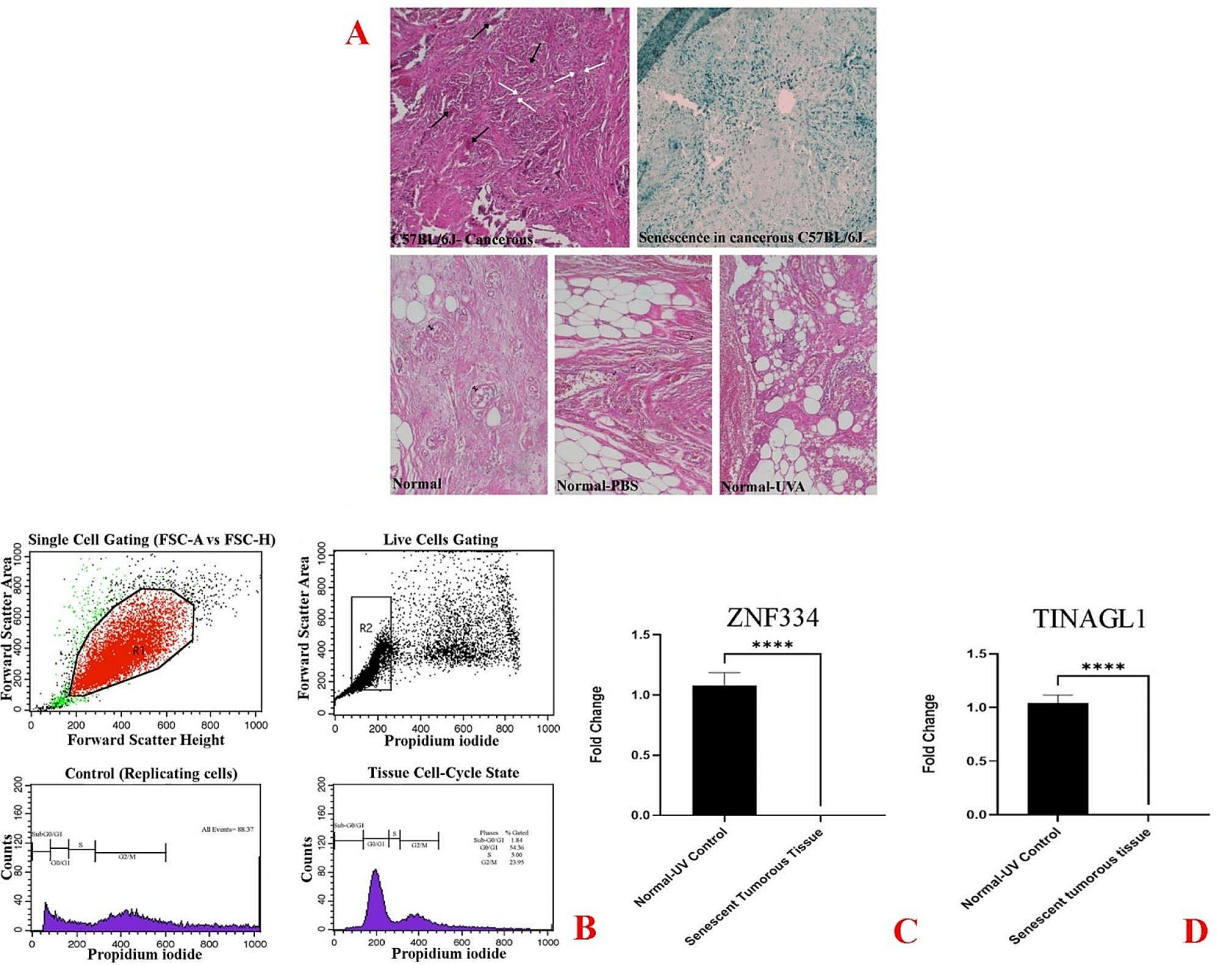
Murine Model of Ultraviolet Radiation Stress-Induced Senescence. The lung SCC tumor-bearing C57BL/6J mice were treated by UVA irradiation for seven consecutive days (2.2 kJ/m^2^ for 30s/day). **A** Histopathology (hematoxylin and eosin-stain) and histochemical detection of SA-β-gal activity of C57BL/6J biopsied tumorous lung. Histopathology: Squamous cell carcinoma was identified in the tumor and shows prominent intercellular bridges (white arrow), keratinization, and a lymphatic invasion pattern (black arrow). Histochemical of SA-β-gal activity: microscopic image of the presence of SA-β-gal activity in C57BL/6J tumorous lung treated by UVA (original magnification, 200×), **B** Flow Cytometry Gating for Single and Live Cells, Quarter 1: Forward Scatter Area vs. Forward Scatter Height; first gate that was used to exclude any doublets and clumps of multiple cells to eliminate skewed results, Quarter 2: Forward Scatter Area vs. Propidium iodide; gate that is used for live cells and gate out obvious debris (as dead cells often have varied areas and heights that can make data look sloppy), once the single cells of interest were the only cells in our analysis, Quarter 3: Control cells stained with propidium iodide show G1, S, and G2/M phase distributions. Quarter 4: The frequency of G1 senescent cells increased dramatically, and the frequencies of S and G2/M phases decreased significantly. Plots were made using ModFit. **C** Significantly decreased *ZNF334* expression in a senescent murine model treated with UVA. qRT-PCR analysis was performed using RNA prepared from a senescent C57BL/6J biopsied tumor lung treated with 2.2 kJ/m^2^ of UVA light. *GAPDH* was used as a reference gene. Data are expressed using the 2^−∆∆Ct^ method. Non-parametric Wilcoxon test was carried out to evaluate statistical differences between groups. Differences were considered significant at *p* < 0.05. **D** Significantly decreased *TINAGL1* expression in a senescent murine model treated with UVA. qRT-PCR analysis was performed using RNA prepared from a senescent C57BL/6J biopsied tumor lung treated with 2.2 kJ/m^2^ of UVA light. *GAPDH* was used as a reference gene. Data are expressed using the 2^−∆∆Ct^ method. Non-parametric Wilcoxon test was carried out to evaluate statistical differences between groups. Differences were considered significant at *p* < 0.05

As cellular senescence has been defined as a mechanism whereby a dividing cell enters a stable cell cycle arrest and is accompanied by increased levels of senescence-associated β-galactosidase (SA-β-Gal) activity [27], the concurrent validation of cell cycle arrest and SA-β-gal activity was used to confirm the senescent phenotype. As depicted in Fig. 8A, treatment at given sub-lethal concentrations of H2O2 (25µM and 50µM) and shown in Figs. 11A and 14A, treatment at given sub-lethal dosage of UVA (8 J/cm^2^ delivered to SK-MES-1 cells and 2.2 kJ/m^2^ delivered to mice lungs) resulted in the observation of a significant number of senescent cells with SA-β-gal staining, whereas increased activity of this lysosomal protein was not observed in the control cells. Moreover, treatment with these senescence-stimuli induced senescence-associated growth arrest (classically defined as an irreversible cell cycle arrest in G1 phase [28]) in all models, which appeared as an increased accumulation of cells in the cell cycle G1 phase. The distribution of cells in the G1, S, and G2 phases of the cell cycle under different treatment conditions was observed in the control group (Figs. 8B, 9A, 10A, 11B, 12A, 13A and 14B). Taken together, these results revealed that the presence of SA-β-gal activity and cell cycle arrest observed in response to H2O2 and UVA irradiation are associated with the induction of senescence in cell lines and C57BL/6J mice lung.

As shown in Figs. 8C & D and 9B&C and 10B&C qRT-PCR analysis revealed that in both experimental groups, the expression level of *ZNF334* in senescent cells (all different cell lines) treated with H2O2 was downregulated, although no significant difference was observed (p value 25µM 0.2188, p value 50µM 0.0938) in SK-MES-1 senescent cells, the difference were significant in senescent NCI-H520 (p value 25µM 0.0078, p value 50µM 0.0078) and NCI-H2170 (p value 25µM 0.0078, p value 50µM 0.0078) cells. Nevertheless, the expression level of *TINAGL1* (p value 25µM 0.0313, p value 50µM 0.0313) has been upregulated significantly in SK-MES-1 senescent cells and significantly downregulated in senescent NCI-H520 (p value 25µM 0.0078, p value 50µM 0.0078) and NCI-H2170 (p value 25µM 0.0078, p value 50µM 0.0078) cells.

Under the same qRT-PCR conditions, the expression levels of *ZNF334* and *TINAGL1* were also measured for both senescent cellular and murine models exposed to UVA, and as depicted in Figs. 11C and D and 12B and C and 13B and C we found that ultraviolet radiation stress-induced senescence led to significant downregulation levels of both *ZNF334* (Cellular model p value 0.0039 _SK−MES−1_, 0.0078 _NCI−H520_, 0.0078 _NCI−H2170_ and Murine model p value < 0.0001) and *TINAGL1* (Cellular model p value 0.0039 _SK−MES−1_, 0.0078 _NCI−H520_, 0.0078 _NCI−H2170_ and Murine model p value < 0.0001) in both model systems.

## Discussion

The low overall survival rate of lung cancer, with particular attention to non-small cell lung cancer (NSCLC), is mainly attributable to metastasis [29]. This declining survival among NSCLC patients is attributed to progression to lymph nodes [4], which is as high as 50% in lung SCC patients, consequently resulting in a 30% shorter survival time compared with other types of NSCLC, such as adenocarcinoma [3].

Application of molecular-based detection of lung SCC metastasis capability is very limited, and its low survival rate is in part due to the lack of metastatic outcome detection biomarkers [4, 30]. Thus, reliable biomarkers are urgently required for the early prediction of lung SCC progression to lymph nodes. With the growing availability of high-throughput technology, the identification of DEGs in disease is one particular focus of the investigation to pinpoint candidate biomarkers [7, 8].

Herein, with the goal of preventing lung SCC metastasis and utilizing bioinformatics technology, we attempted to identify the potential biomarkers of the lymph progression based on the expression levels of highly connected DEGs to lung SCC lymph node metastasis. In the first part of the study, a total of specifically 950 DEGs, comprising 611 up-regulated and 339 down-regulated genes, were identified specific to N + samples by comparison of gene expression profiles between two biological states of N + vs. normal and N0 vs. normal. Second, the study of the PPI network provided an insight into a subnetwork with highly connected genes that were significant in mitochondrial respiratory electron transfer pathways. Within the strongest subnetwork in the N+-specific DEGs, there are five genes that encode subunits of mitochondrial NADH: ubiquinone oxidoreductase (complex I). These genes consist of *NDUFC1*, *NDUFB2*, *NDUFB6*, *NDUFB7*, and *NDUFS1*, with *NDUFS1* being the largest subunit of complex I.

Briefly, NADH-ubiquinone oxidoreductase is the largest complex of the mitochondrial electron transport chain, composed of 45 subunits, generating, together with proton-pumping complexes III and IV, the electrochemical proton gradient required for ATP synthesis [3].

The activity of Complex I, an enzyme complex involved in the electron transport chain within the inner mitochondrial membrane, can have contrasting effects on migration, invasion, and metastasis in different types of cancers. In general, inhibiting Complex I activity while generating reactive oxygen species (ROS) promotes these cancer-related processes [31]. However, recent research has shown that the overexpression of certain subunits of Complex I is associated with migration and invasion of non-small-cell lung cancer cells [32]. Additionally, lung cancer cells that are resistant to cisplatin treatment exhibit high Complex I activity, increased mitochondrial transmembrane potential, elevated ATP content, and enhanced migration and invasion compared to non-resistant cells [32]. These conflicting observations regarding Complex I activity and its effects on migration, invasion, and metastasis can be attributed to cancer-specific differences but are most likely influenced by the extent of Complex I inhibition, which ultimately determines its pro- or anti-tumorigenic effects [33, 34]. Interestingly, in our study, we also found increased expression of complex I components in the N + status of lung SCC. Importantly, *NDUFC1* has the highest gene expression ratio in the N + compared to the N0 status of lung SCC, and interestingly, in line with our findings, it is associated with a higher risk of lymphatic metastasis, a higher proportion of positive lymph nodes, and a more advanced tumor stage in gastric cancer [35] and hepatocellular carcinoma [36].

Other proteins of our founded subnetwork, including *COX4I1*, *UQCRC2*, *ATP5J (ATP5PF)*, and *ENSP00000400168* (ATP5MF-PTCD1), and *UQCRQ* also play a role in mitochondrial ATP synthesis; they are also involved in tumor initiation, growth, and metastasis [37].

In addition, we obtained the top ten hub genes for N+-specific lung SCC DEGs that showed dependency on the RNA polymerases (Pol I, Pol II, and Pol III) transcription machinery activity and that seem to be consistent with the acquisition of genetic alterations by cancer cells in regulatory layers of pre-ribosomal RNA (rRNA) and transfer RNA (tRNA) transcription that play significant roles during tumor progression and metastasis. In healthy cells, transcription by polymerases is restrained by tumor suppressors; however, such restraints are compromised during cell transformation, resulting in increases in rRNA and tRNA expression that strongly impact metastasis [38, 39].

While the mentioned genes were not identified as a gene set associated with lung SCC specifically, each of these genes has been previously verified in lung cancer studies individually, so we applied strict filtering to focus on the more interesting and novel genes among the 15 top down- and up-regulated genes from hundreds of N+-specific DEGs for verification by experimental approaches.

Our findings indicated more novel genes within the top down-regulated genes associated with metastatic dissemination, while certain top-up-regulated genes had been well-studied in the literature. Finally, it resulted in the selection of two representative genes (*ZNF334* and *TINAGL1*) from the down-regulated genes to be further analyzed in our study.

*ZNF334* is a newly identified member of Zinc Finger Proteins (ZNFs), and over the last few decades, increasing evidence has supported key roles for ZNFs in cancer metastasis through regulating transcription of downstream genes, which are involved in proliferation, apoptosis, senescence, migration, and invasion [40]. The second selected down-regulated gene is *TINAGL1*, an extracellular matrix protein that promotes integrin-mediated cell adhesion, whose expression is dependent on Sec. 23 homolog A (SEC23A), an essential component of coat protein complex II vesicles that is indispensable for ECM protein secretion [41].

Our bioinformatics analyses revealed that *ZNF334* was downregulated in lung SCC-LN metastases. To validate the results from the bioinformatics analyses, the expression of the *ZNF334* gene was analyzed in vitro, ex vivo, and in vivo in N + lung squamous cell carcinoma cells and tissues by qRT-PCR. In agreement with the bioinformatics results, LNM lung SCC tissues (ex vivo and in vivo) and three different cell lines exhibited significantly downregulated expression of *ZNF334* compared with normal tissues and cell lines. These results were also consistent with other studies findings that reported downregulated expression of *ZNF334* in various metastatic cancers. Cheng et al. reported decreased expression of *ZNF334* in breast cancer tissues and cell lines and showed that re-expression of *ZNF334* in TNBC cell lines could suppress their growth and metastatic capacity [42]. Yang et al. detected the downregulation of *ZNF334* gene expression in the occurrence of colorectal cancer and defined it as a molecular marker for the early diagnosis of colorectal cancer [40]. Similarly, Sun et al. clarified the effect of the *ZNF334* deletion on the occurrence of hepatocellular carcinoma and described it as a molecular marker for liver cancer early diagnosis [43].

Although our mined bioinformatics results implicated that the downregulated *TINAGL1* gene was mainly involved in the N + status of lung SCC, an inconsistency between in vitro (SK-MES-1cell line) and ex vivo results for the *TINAGL1* gene and those obtained using bioinformatics analysis was observed. Nonetheless, the validated result from the NCI-H520 and NCI-H2170 cell lines and in vivo experiment in mice was in agreement with the bioinformatics result, which in the case of animal model has been attributed to differences between the human and mouse genomes. Intriguingly, the obtained results from our in vitro and ex vivo validation were in concordance with other studies that reported *TINAGL1* as a Sec23a-dependent metastasis suppressor being upregulated in metastatic tumors [44]. In the present study, since using a higher number of biological replicates (six independent biological replicates) increased the analysis accuracy and reliability and the quality and quantity of our human tissue samples were acceptable for genomic analysis, we suggested that the significantly upregulation of *TINAGL1* plays an important role in the pathological process of lung SCC lymph node progression. Sun et al. also determined the *TINAGL1* up-regulation in human hepatocellular carcinoma tissues and explained that *TINAGL1* overexpression promotes hepatocellular carcinogenesis and metastasis via the TGF-β/Smad3/VEGF axis [45]. Moreover, Shan et al. highlighted the increased expression of *TINAGL1* in gastric cancer tumor tissues and its association with gastric cancer tumor growth and metastasis [46]. In contrast, another study that also focused on the role of *TINAGL1* in cancer metastasis showed lower *TINAGL1* expression correlates with advanced triple-negative breast cancer tumor stages, reduced disease-free survival, and distant metastasis-free survival [47]. Similarly, our in vivo and in vitro quantitative analysis in two NCI-H520 and NCI-H2170 cell lines revealed a significantly decreased expression level of *TINAGL1* compared to normal lung SCC.

To explain why human *TINAGL1* gene expression differs from mouse, a better understanding of the differences between the human and mouse genomes has clarified that genes involved in intracellular processes such as RNA processing and chromatin organization (transcription factors binding to promoter regions like the *ZNF334* gene) tend to have a similar gene expression pattern between humans and mice (highly conserved), whereas genes involved in extracellular matrix and cellular adhesion (distant regulatory elements like the *TINAGL1* gene) and signaling receptors are less conserved (lineage-specific) [48–50].

Interestingly, in our study, although the positive role of *ZNF334* in lung SCC lymph node progression was verified and was quite gratifying, in terms of diagnostic performance, it could not gain a significant diagnostic value in ROC analysis (AUC 0.68). While the area under the ROC curve of the *TINAGL1* gene exceeds 0.8, suggesting that this gene can discriminate between N + and N0 lung SCC.

Finally, we evaluated the senescence-induced alterations in *ZNF334* and *TINAGL1* gene expression in our established cellular and murine senescent model systems. In summary, we observed an increasing and decreasing expression level of *TINAGL1* upon exposure to hydrogen peroxide (in SK-MES-1 cell line and NCI-H520 and NCI-H2170 two lines, respectively) and decreasing expression level upon exposure UVA irradiation stimuli in all three cell lines and murine model, that have been used to promote cellular senescence. It has been shown that increases in hydrogen peroxide and its related pathological changes in reactive oxygen species levels lead to excess ECM production [51, 52]. While UV-induced ROS downregulates the transforming growth factor beta (TGF-β) pathway, which reduces extracellular matrix protein production [53–55]. In addition, PPARγ activity occurs as a result of UV-generated reactive oxygen species, which are accompanied by inhibition of the expression of ECM genes [56, 57]. Senescence is associated with both changes in the ECM components. These changes alter cellular behavior and potentially drive cells to become senescent [58].

However, we observed a drastic decrease in expression of *ZNF334* under senescence conditions. As mentioned above, *ZNF334* is a member of the C2H2 zinc finger family. Recently, proteins containing C2H2 ZnFs have been identified as key regulators of telomere length in mammalian cells [59]. Loss of telomere protection can act as a barrier to tumorigenesis [60]. It seems that repression of *ZNF334* under induced senescence conditions might cause complete telomere deprotection and contribute to senescence-related pathologies.

Our findings on *ZNF334* and *TINAGL1* gene expression alterations and their effect as a negative regulator of cellular senescence are supported by evidence that shows zinc finger proteins’ genes dysregulation are involved in telomere maintenance and genome integrity in cancer [61] or aberrant extracellular matrix proteins potentially influence the senescent phenotype [62, 63]. In addition, the role of other ZNF aberrations in cellular senescence bypass has been confirmed in some experimental studies. For instance, Villot et al. recently showed that zinc-finger protein 768 (ZNF768) controls cell fate and that its aberrant expression allows cells to bypass senescence [64]. Xie et al. found that *ZNF277* inhibits cellular senescence by repressing *p21WAF1* expression in human colon cancer cells [65]. Gao et al. suggested that the overexpression of *Zfp637* markedly increases *mTERT* expression and telomerase activity by maintaining telomere length and inhibiting cellular senescence [66]. Xiang et al. demonstrated that *GATA4* is silenced in hepatocellular carcinoma and that restoration of *GATA4* expression induces cellular senescence through regulating nuclear factor-κB [67]. On the other hand, several experiments showed extracellular matrix proteins’ role in cellular senescence. Shelton et al. suggested that retinal pigment epithelial cells (RPE) can undergo senescence by remodeling *COL1A1*, *COL1A2*, and *COL3A1* mRNA expression levels [68]. Schnabl et al. showed that in the liver, the reduction in collagen type 1 mRNA expression and protein levels resulted in hepatic stellate cells becoming senescent [69]. Likewise, it showed that a reduction in the *COL3A1*, *COL4A1*, *COL4A2*, and *COL5A1* gene expression induced senescence in hepatic stellate cells [70].

Generally, our bioinformatics and experimental analyses identified several important genes involved in mitochondrial electron transport complex I and ATP synthesis, transcription machinery components, and senescence escape-specific genes that may play a role in the early detection of lung SCC metastasis at lymph nodes. Some suggestions for future research include that the detected DEG pool in this study is not limited to the analysis of the *ZNF334* and *TINAGL1* genes, and other interesting biomarkers with common functions can be identified.

## Conclusion

The results of our bioinformatics analyses showed that mitochondrial electron transport chains and transcription machinery-related gene panels could be used to distinguish lymph node metastatic lung SCC from benign tumors. Collectively, our integrated bioinformatics-experimental analyses results highlight the role of *TINAGL1* as biomarker for distinguish lymph node metastatic lung SCC from benign tumors and the potential for *ZNF334* as a generalizable gene to senescence bypass during lung squamous cell carcinoma progression to lymph nodes.

### Electronic supplementary material

Below is the link to the electronic supplementary material.

Supplementary Material 1

Supplementary Material 2

Supplementary Material 3

Supplementary Material 4

Supplementary Material 5

Supplementary Material 6

## Data Availability

Dataset analyzed during the current study (TCGA-LUSC dataset) were previously generated and are available in the TCGA repository (https://portal.gdc.cancer.gov/).

## Abbreviations

SCC: Squamous cell carcinoma
LNM: lymph node metastasis
RNA-seq: RNA sequencing
TCGA: The cancer genome database
CPM: Counts per million
TMM: Trimmed mean of m-values
OD: Optical density
cDNA: Complementary DNA
qPCR: Quantitative PCR
ZNFs: Zinc finger proteins
UV: Ultraviolet
H2O2: Hydrogen peroxide
SA-β-gal: Senescence-associated beta-galactosidase
PI: Propidium iodide
ROC: Receiver operating characteristic
AUC: Areas under the curve
TGF-β: Transforming growth factor beta
NSCLC: Non-small cell lung cancer
ZNFs: Zinc finger proteins
STRING: Search tool for retrieval of interacting genes
PPI: Protein–protein interaction
MCODE: Multi-contrast delayed enhancement
GO: Gene ontology
BP: Biological process
MF: Molecular function
CC: cellular component
ROS: Reactive oxygen species
rRNA: pre-ribosomal RNA
tRNA: transfer RNA
