# Supplementary material for: Implications of ZNF334 gene in lymph node metastasis of lung SCC: potential bypassing of cellular senescence
Source: J Transl Med. 2024 Apr 18;22:372. doi: 10.1186/s12967-024-05115-9 (PMC11025273; doi:10.1186/s12967-024-05115-9)
Supplement: Supplementary file 2 — Supplementary Material 2 [file 12967_2024_5115_MOESM2_ESM.docx]

Filtered top up- regulated genes

| Names | N+ vs Normal (logFC) | N+ vs Normal (FDR) |
| --- | --- | --- |
| *H19*  *CCDC190*  *ABCA4*  *CPLX2*  *EPCAM*  *CLDN8*  *KRT8*  *HAPLN1*  *PLA2G2D*  *MYB*  *WSCD2*  *PCSK1N*  *KLK14*  *NRXN2*  *IGHV4-61*  *GPR63* | 1.016719654  0.835646309  0.780391109  0.750312582  0.703648042  0.699170111  0.696651061  0.65661086  0.589692123  0.585108213  0.547334849  0.539913247  0.530609638  0.517771518  0.517455944  0.508228816 | 0.016808083  0.01796637  0.031428352  0.016721554  0.000479728  0.031388366  0.000229425  0.000510146  0.026084706  0.019848571  0.029492719  0.010256948  0.011797622  0.019596158  0.049669721  0.000641251 |

Filtered top down-regulated genes

| Names | N+ vs Normal (logFC) | N+ vs Normal (FDR) |
| --- | --- | --- |
| *KDM5D*  *USP9Y*  *TSPAN1*  *SYT8*  *SNCG*  *TINAGL1*  *PADI2*  *ZIK1*  *GGT6*  *MDGA1*  *ZNF334*  *PDPN*  *CCDC8*  *FCGBP*  *TM4SF1*  *PMEPA1*  *KLF8*  *INPP1*  *PRAG1*  *MMP2*  *GABRB3*  *NYNRIN*  *IRAG2*  *DCLK1* | -0.822655619  -0.820417227  -0.775142614  -0.772753709  -0.737831404  -0.687630634  -0.657953184  -0.639690407  -0.628763369  -0.6072013  -0.570167591  -0.566828425  -0.560398478  -0.557793791  -0.548260917  -0.544318576  -0.532047001  -0.519034309  -0.51733891  -0.515535877  -0.513515916  -0.511058818  -0.503892087  -0.503030108 | 0.031216885  0.019388416  0.011323949  0.003488192  0.01141025  0.009304419  0.004808778  0.000870969  0.007888226  0.027808412  0.006742199  0.023557126  0.039419114  0.041961341  0.011452282  0.039259084  0.008775735  0.00173922  0.000365251  0.030263993  0.040616508  0.024954142  0.038616114  0.018969923 |
