# Supplementary material for: Implications of ZNF334 gene in lymph node metastasis of lung SCC: potential bypassing of cellular senescence
Source: J Transl Med. 2024 Apr 18;22:372. doi: 10.1186/s12967-024-05115-9 (PMC11025273; doi:10.1186/s12967-024-05115-9)
Supplement: Supplementary file 6 — Supplementary Material 6 [file 12967_2024_5115_MOESM6_ESM.docx]

***COX4I1***

Denisov EV, Schegoleva AA, Gervas PA, Ponomaryova AA, Tashireva LA, Boyarko VV, Bukreeva EB, Pankova OV, Perelmuter VM. Premalignant lesions of squamous cell carcinoma of the lung: The molecular make-up and factors affecting their progression. Lung Cancer. 2019 Sep;135:21-28. doi: 10.1016/j.lungcan.2019.07.001.

***NDUFS1***

Su CY, Chang YC, Yang CJ, Huang MS, Hsiao M. The opposite prognostic effect of NDUFS1 and NDUFS8 in lung cancer reflects the oncojanus role of mitochondrial complex I. Sci Rep. 2016 Aug 12;6:31357. doi: 10.1038/srep31357.

***NDUFC1***

Xu L, Chen X, Jiang H, Xu J, Wang L, Sun Y. NDUFC1 Is Upregulated in Gastric Cancer and Regulates Cell Proliferation, Apoptosis, Cycle and Migration. Front Oncol. 2021 Dec 13;11:709044. doi: 10.3389/fonc.2021.709044.

***NDUFB2***

Li B, Zhu L, Lu C, Wang C, Wang H, Jin H, Ma X, Cheng Z, Yu C, Wang S, Zuo Q, Zhou Y, Wang J, Yang C, Lv Y, Jiang L, Qin W. circNDUFB2 inhibits non-small cell lung cancer progression via destabilizing IGF2BPs and activating anti-tumor immunity. Nat Commun. 2021 Jan 12;12(1):295. doi: 10.1038/s41467-020-20527-z.

***UQCRC2* and *UQCR Q***

Herst PM, Carson GM, Eccles DA, Berridge MV. Bioenergetic and Metabolic Adaptation in Tumor Progression and Metastasis. Front Oncol. 2022 Mar 17;12:857686. doi: 10.3389/fonc.2022.857686.

***NDUFB6***

Lo KC, Stein LC, Panzarella JA, Cowell JK, Hawthorn L. Identification of genes involved in squamous cell carcinoma of the lung using synchronized data from DNA copy number and transcript expression profiling analysis. Lung Cancer. 2008 Mar;59(3):315-31. doi: 10.1016/j.lungcan.2007.08.037.3

***ATP5J* (*ATP5PF*)**

Lu J, Li Y, Gong S, Wang J, Lu X, Jin Q, Lu B, Chen Q. Ciclopirox targets cellular bioenergetics and activates ER stress to induce apoptosis in non-small cell lung cancer cells. Cell Commun Signal. 2022 Mar 24;20(1):37. doi: 10.1186/s12964-022-00847-x.

***NDUFB7***

Hertweck KL, Vikramdeo KS, Galeas JN, Marbut SM, Pramanik P, Yunus F, Singh S, Singh AP, Dasgupta S. Clinicopathological significance of unraveling mitochondrial pathway alterations in non-small-cell lung cancer. FASEB J. 2023 Jul;37(7):e23018. doi: 10.1096/fj.202201724RR.

***ENSP00000400168* (*ATP5MF-PTCD1*)**

Yang Y, Zhang Y, Miao L, Liao W, Liao W. LncRNA PPP1R14B-AS1 Promotes Tumor Cell Proliferation and Migration via the Enhancement of Mitochondrial Respiration. Front Genet. 2020 Nov 11;11:557614. doi: 10.3389/fgene.2020.557614.

***POLR2F***

Antonacopoulou AG, Grivas PD, Skarlas L, Kalofonos M, Scopa CD, Kalofonos HP. POLR2F, ATP6V0A1 and PRNP expression in colorectal cancer: new molecules with prognostic significance? Anticancer Res. 2008 Mar-Apr;28(2B):1221-7.

***RPL19***

Kuroda K, Takenoyama M, Baba T, Shigematsu Y, Shiota H, Ichiki Y, Yasuda M, Uramoto H, Hanagiri T, Yasumoto K. Identification of ribosomal protein L19 as a novel tumor antigen recognized by autologous cytotoxic T lymphocytes in lung adenocarcinoma. Cancer Sci. 2010 Jan;101(1):46-53. doi: 10.1111/j.1349-7006.2009.01351.x.

***RPL13A***

Hellwig B, Madjar K, Edlund K, Marchan R, Cadenas C, Heimes AS, Almstedt K, Lebrecht A, Sicking I, Battista MJ, Micke P, Schmidt M, Hengstler JG, Rahnenführer J. Epsin Family Member 3 and Ribosome-Related Genes Are Associated with Late Metastasis in Estrogen Receptor-Positive Breast Cancer and Long-Term Survival in Non-Small Cell Lung Cancer Using a Genome-Wide Identification and Validation Strategy. PLoS One. 2016 Dec 7;11(12):e0167585. doi: 10.1371/journal.pone.0167585.

***MTOR***

Yu T, Zhao Y, Hu Z, Li J, Chu D, Zhang J, Li Z, Chen B, Zhang X, Pan H, Li S, Lin H, Liu L, Yan M, He X, Yao M. MetaLnc9 Facilitates Lung Cancer Metastasis via a PGK1-Activated AKT/mTOR Pathway. Cancer Res. 2017 Nov 1;77(21):5782-5794. doi: 10.1158/0008-5472.CAN-17-0671.

***NEDD8***

Li L, Kang J, Zhang W, Cai L, Wang S, Liang Y, Jiang Y, Liu X, Zhang Y, Ruan H, Chen G, Wang M, Jia L. Validation of NEDD8-conjugating enzyme UBC12 as a new therapeutic target in lung cancer. EBioMedicine. 2019 Jul;45:81-91. doi: 10.1016/j.ebiom.2019.06.005.

***RPSA***

Wu Y, Tan X, Liu P, Yang Y, Huang Y, Liu X, Meng X, Yu B, Wu M, Jin H. ITGA6 and RPSA synergistically promote pancreatic cancer invasion and metastasis via PI3K and MAPK signaling pathways. Exp Cell Res. 2019 Jun 1;379(1):30-47. doi: 10.1016/j.yexcr.2019.03.022.

***POLR3B***

Musolf AM, Moiz BA, Sun H, Pikielny CW, Bossé Y, Mandal D, de Andrade M, Gaba C, Yang P, Li Y, You M, Govindan R, Wilson RK, Kupert EY, Anderson MW, Schwartz AG, Pinney SM, Amos CI, Bailey-Wilson JE. Whole Exome Sequencing of Highly Aggregated Lung Cancer Families Reveals Linked Loci for Increased Cancer Risk on Chromosomes 12q, 7p, and 4q. Cancer Epidemiol Biomarkers Prev. 2020 Feb;29(2):434-442. doi: 10.1158/1055-9965.EPI-19-0887.

***SEC13***

Vicary GW, Roman J. Targeting the Mammalian Target of Rapamycin in Lung Cancer. Am J Med Sci. 2016 Nov;352(5):507-516. doi: 10.1016/j.amjms.2016.08.014.

***RPS15A***

Ning Q, Pang Y, Shao S, Luo M, Zhao L, Hu T, Zhao X. MicroRNA-147b suppresses the proliferation and invasion of non-small-cell lung cancer cells through downregulation of Wnt/β-catenin signalling via targeting of RPS15A. Clin Exp Pharmacol Physiol. 2020 Mar;47(3):449-458. doi: 10.1111/1440-1681.13203.

***UBQLN1***

Shah PP, Saurabh K, Kurlawala Z, Vega AA, Siskind LJ, Beverly LJ. Towards a molecular understanding of the overlapping and distinct roles of UBQLN1 and UBQLN2 in lung cancer progression and metastasis. Neoplasia. 2022 Mar;25:1-8. doi: 10.1016/j.neo.2021.11.010.
